# Ecological patterns in anchialine caves

**DOI:** 10.1371/journal.pone.0202909

**Published:** 2018-11-07

**Authors:** Fernando Calderón-Gutiérrez, Carlos A. Sánchez-Ortiz, Leonardo Huato-Soberanis

**Affiliations:** 1 Departamento Académico de Ciencias Marinas y Costeras, Universidad Autónoma de Baja California Sur, La Paz, Baja California Sur, México; 2 Programa de Ecología Pesquera, Centro de Investigaciones Biológicas del Noroeste (CIBNOR), La Paz, Baja California Sur, México; Universita degli Studi di Genova, ITALY

## Abstract

Anchialine caves are characterized by high levels of endemism and extreme conditions. However, few ecological studies have been conducted in these ecosystems. This study integrates biotic and abiotic parameters of two sets of cave systems with contrasting high and low species richness. Seven ecological patterns are used to explain the expected species richness and density in an anchialine cave. In addition, the population size for conspicuous macrofauna was estimated. The high impact that single-events have on anchialine fauna are also reported. These findings reinforce the conclusions of previous studies of the high extinction risk of anchialine cave fauna, and substantiate the necessity of *ad hoc* conservation strategies for anchialine caves.

## Introduction

Underwater caves can be freshwater, marine or anchialine (i.e., “tidally-influenced subterranean estuary located within crevicular and cavernous karst and volcanic terrains that extends inland to the limit of seawater penetration” [1,2]) [1,3,4]. While different types of underwater caves have some ecological similarities, some specific patterns could exist. Underwater caves have characteristic fauna with high levels of micro-endemism (species known from only one or two caves) [5–7], relict species [8], and fauna related to those from the deep sea [9,10]. As a result, they have been identified as natural laboratories [4,11–13], where the process of speciation in cave fauna arises from adaptation to unique conditions such as the lack of light, low energy availability, and semi-isolation [4,14].

Despite the importance of identifying underwater cave fauna, caves are still one of the least studied environments [15]. To date, most cave studies have focused on taxonomy [3,8,16–25], while ecological studies primarily concern marine caves [3,26–29]. Similar investigations of anchialine caves are almost nonexistent, concerning only a few species or caves of interest [30–33]. As a result, there is a limited understanding of the ecological function within anchialine cave environments [34,35].

The main factors involved in the stratification of the fauna among and within anchialine caves remain largely understudied [34,35]. In dry caves, differences have been reported between caves longer than 200m and shorter ones [4]. Marine caves are also known to support higher specific richness than anchialine caves; the same pattern is likely to happen in anchialine caves with direct connection to the sea compared with those with indirect connection (i.e. marine water enters the cave through fissures and pores of the rock) [1,20,26,28,35,36]. Several other factors have been also reported to influence the community structure in caves, such as depth, location of the halocline, and food availability [4,35].Since most anchialine species are characterized by low population densities and high levels of endemism [4,27,32,37,38], they are especially vulnerable to the threat of extinction as a result of anthropogenic pressures [26,27,29,36,39–41]. As consequence, it is essential to understand and protect such fragile anchialine ecosystems to prevent loss in biodiversity, and potentially permanent alteration of their community structure and function.

Due to its karstic nature, low elevation and relatively flat topography, the Yucatan Peninsula has one of the highest densities, as well as the longest explored anchialine cave systems in the world [20,42–45]. In particular, the anchialine caves of Cozumel Island have a high biodiversity [19,20,46], including numerous stygobitic (i.e., aquatic, obligate cave-dwellers) species [5,7,47]. Additionally, Cozumel’s caves vary in their geomorphological features (e.g., size, depth, distance to the sea) [48], making them an ideal place to detect ecological patterns without geographical bias that may result from the comparison of anchialine caves located in multiple locations.

The present study, conducted from January 2011 to July 2016, compared the species richness, population size and community structure of the macroorganisms inhabiting four anchialine caves on Cozumel Island, as well as environmental parameters affecting the distribution of the fauna. This data was used to identify seven ecological patterns related with the cave morphology and other abiotic factors influencing the expected biodiversity in anchialine caves, which are thoroughly described in the discussion.

## Materials and methods

### Ethics statement

A non-invasive visual survey was carried out by scientific cave divers using technical diving techniques, requiring specialized training in order to be carried out safely and to avoid damage to the caves and its fauna. All samples collected fall under a permit from the Comisión Nacional de Acuacultura y Pesca (CONAPESCA). Samples of no more than three individuals per morphotype were collected and identified. All organisms were relaxed before being fixed.

### Study area

**Fig 1:**
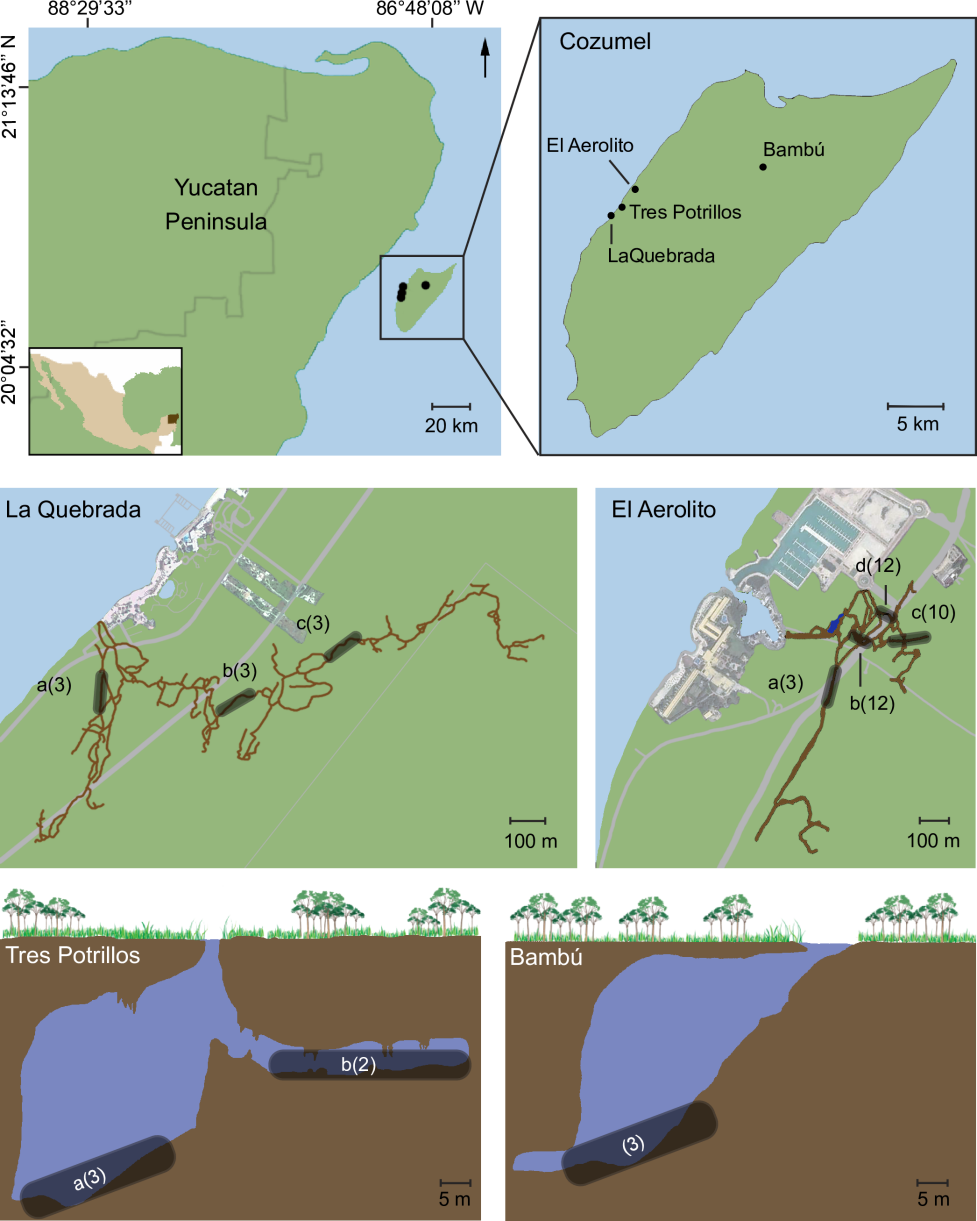
Study area. Maps of the four anchialine caves displaying the biological census sites as shaded regions indicated by a letter (identical letters between caves not being related), the number of replicates is in brackets.

This study was conducted in two pairs of anchialine caves with similar geomorphological features (e.g. depth, length, type of connection with the sea) to compare the degree of influence of the morphology of an anchialine cave on the community structure. The selected caves are located on Cozumel Island, Quintana Roo, Mexico (Fig 1): La Quebrada, El Aerolito, Tres Potrillos and Bambú. La Quebrada and El Aerolito have a direct connection to the sea, are shallow (average depth of 6 and 12m, maximum of 9.7 and 27m, respectively), and long (9.2 and 18km, respectively). The principal sediments are mud and clay, while the presence of stalactites and stalagmites (which only form in air) indicate that the caves have been dry in the past. Tres Potrillos and Bambú are inland, bell shaped cenotes (i.e. “steep-walled natural well that extends below the water table” [49]) lacking direct connection with the sea. They are approximately 60m long and are 38m and 52m deep, respectively [20,48]. Dominant sediments are silt and organic matter accumulated from the adjacent jungle. In addition, both caves contain significant levels of hydrogen sulfur. Sites of each anchialine cave were designated with a letter, identical letters between caves are not related.

La Quebrada (Fig 1, S1 Table), also known as Chankanaab (20°26’N, 86°59’W), has 8 cenotes and four main entrances: Km 1, Roca Bomba, Cilpa and C1. The water layers across the halocline, at 4m depth, are mesohaline above (salinity <17) and mixoeuhaline below (salinity >30) [48,50]. The cave ceiling of La Quebrada has a thickness of less than 1.5m to the surface of the ground (measured with a diving computer). Biological census sites were located at increasing distance for the sea: site “a” (216m from the sea) at 4.5 to 5.2m depth, site “b” (526m from the sea) at 7.2 to 7.5m depth; and site “c” (833m from the sea) at 6.5 to 7.2m depth.

El Aerolito (Fig 1, S1 Table) has two entrances: the main one at cenote El Aerolito del Paraíso (20°27’N; 86°58’W), and “La Caleta” (240m away), which opens directly to the sea at a small boat harbor. The halocline lies at 7m depth with a change from mesohaline to mixoeuhaline water. A sulfidic layer (at lower concentration than in Tres Potrillos and Bambú) [20,48] is located at the end of the 9m depth main passage. Biological census sites were located along the cave as follows: site “a” was located in a lateral passage, starting at 225m from the cenote entrance, at 10.9 to 12m depth; site “b” was located in the main passage, starting at 285m from the cenote entrance, at 9 to 10m depth; site “c” was located at the end of the main passage at 9 to 11.8m depth; and site “d” was the only one with a rocky bottom and completely mixoeuhaline water, at 17 to 18.4m depth.

Tres Potrillos (Fig 1, S1 Table) is located at 258m from the coastline, has a single entrance (20°27’N; 8600B003059’W), with a halocline at 12m depth. A secondary passage extends off the main chamber at 16m depth [48]. Tres Potrillos has high levels of tannic acid from the surface to 5m depth. Biological census sites were located along the cave as follows: site “a” was located in the deep zone at 36m depth; and site “b” in the secondary passage at 16m depth.

Bambú (Fig 1, S1 Table) is the only known anchialine cave in the north central region of Cozumel Island at 5,263m from the coastline (20°29’N; 86°52’W), with a halocline at 42m depth. Bambú has tannic acid in the first several meters and a high concentration of sulfuric acid from the surface to 21m depth, which limits visibility to 5m [48], and has microbial mats on the walls. There was a single biological census site, at 45m depth.

### Abiotic information

Temperature was measured in 5-minute intervals from January 2015 to June 2016 with HOBO Pendant UA-002-64 Onset sensors. The measurements where only interrupted in October 2015 during one to two days for battery replacement. In El Aerolito, four HOBO sensors were set: at the cave entrance below the halocline (5m depth); at 100m from the entrance above the halocline (4m depth); at the end of the main passage (site “c”) below the halocline (11m depth); and at the beginning of site “d” (18m depth). In La Quebrada, HOBO sensors were placed below the halocline at the beginning of site “a” (5m depth), and a second (installed on October 26, 2015) at the end of the site “c”. In Tres Potrillos and Bambú, HOBO sensors were set at 27m depth, in the mixoeuhaline and mesohaline layer, respectively.

Precipitation data was obtained from February 2015 to July 2016 at 10-minute intervals from the Cambio Climático en Áreas Naturales Protegidas platform (“Climate Change in Protected Natural Areas”—http://cambioclimatico.conanp .gob.mx/emas.php). Data was collected by the meteorological station of the Servicio Metereológico Nacional (National Metereological Service) QR04 (NOAA 15B5F622), located near the center of Cozumel Island.

Abiotic data recorded at each census site included depth, distance of the halocline from the bottom (difference between bottom and halocline depth, sites lacking a halocline were recorded as 100m in order to differentiate those during the analysis) with a Mares Nemo Sport dive computer. The length of each cave, distance of census sites to the coastline, and the distance to the nearest entrance were obtained from surveyed maps [48]. The percentage of organic matter in the sediment was determined using the Walkley-Black’s technique [51]. Substratum type was categorized in accordance to the predominant type (soft vs hard substratum), and the type of connection to the sea (direct or indirect). All the information is available in S1 Table.

### Macrofauna community

Macroorganisms were collected using cave diving techniques from January 2011 to July 2016. Up to 28 dives were completed per anchialine cave to reach the asymptote of the species accumulation curve (S1 Fig). Collection was conducted by hand, placing individual organisms in sealed plastic bags to avoid potential mixture of taxonomic characteristics. Up to three organisms were collected per operational taxonomic unit (OTU), to limit potential impacts to natural populations. Organisms were relaxed with magnesium chloride or menthol (previously dissolved in water from the cave), until they did not respond to physical stimuli. All organisms were preserved in 70% ethanol. Recorded species in scientific literature were taken into account in order to have the best representation of the macrofauna in the study area [5,20,47]. The taxonomic status of the reported species was validated using WoRMS [52]. Organisms behavior and underwater conditions (e.g. current) were recorded on video with a Go Pro camera 4.

Density of each conspicuous species was obtained at each census site by 15x1m belt transects per triplicate, when possible. Due to cave morphology, transects in La Quebrada and El Aerolito were connected to the permanent guideline (a line used for navigation, enabling cave divers to safely exit the cave). Transects in Tres Potrillos and Bambú were parallel to the permanent guideline. For safety reasons, transects in Bambú were of 10x1m. At the site “b” in Tres Potrillos, transects were completed in duplicates, due to the limited size of this section of the cave (Fig 1). In La Quebrada, nine transects (three per site) were performed; 37 in El Aerolito (site a = 3, b = 12, c = 10, d = 12), five in Tres Potrillos (site a = 3, b = 2), and three in Bambú, for a total census area of 795m^2^ (Fig 1). Biological surveys were performed in January 2015 for all the caves. In addition, El Aerolito was surveyed in July 2011, January 2012 and July 2012, as part of a continuing monitoring program [32].

Comparisons of the density and macrofaunal diversity between caves and between sites were made to detect ecological similarities. Kruskal-Wallis analysis was performed using STATISTICA 8.0, since the data was not normal nor homoscedastic in accordance to the Kolmogorov-Smirnov and Barlett tests (α = 0.05). *A posteriori* analysis was used with multiple comparisons among treatment means for Kruskal-Wallis. To have a better support of the conclusions, a non-metric multidimensional scaling analysis (nMDS–using the Euclidean distance), and an ordination analysis using the Bray-Curtis index were performed on Past 3.02ª based on the density of the recorded species.

### Population estimation

Anchialine caves were divided into distinctive regions, based on the abiotic and biotic data. The total area of each cave was estimated by using information from their cave maps [48]. The average cave width was measured during fieldwork in La Quebrada and El Aerolito, since maps only provided cave lengths. Thus, total areas were 52,826 m^2^ in El Aerolito, 39,353 m^2^ in La Quebrada, and 496 m^2^ in Tres Potrillos.

Subsequently, species population size per region was estimated by extrapolating the average density and standard deviation to the area of the corresponding region. Finally, the regional population size estimates from the same cave were added to calculate the species population size estimate.

## Results

### Abiotic parameters

**Fig 2:**
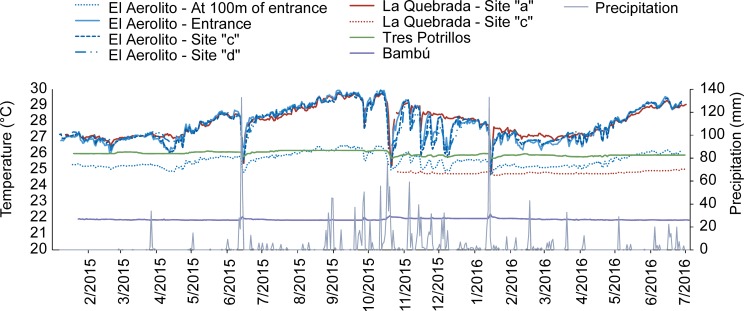
Temperature recorded with HOBO sensors inside the anchialine caves. With the exception of the sensors in El Aerolito at 100m from the entrance and in Bambú, all measurements were in the mixoeuhaline water layer. Precipitation recorded in Cozumel Island is also shown (meteorological station of Servicio Metereológico Nacional QR04—NOAA 15B5F622).

Temperature patterns of the mixoeuhaline waters from anchialine caves were dependent on the nature of the connection with the sea (Fig 2, S2 Table). Caves with direct connection to the sea exhibited similar temperatures in the mixoeuhaline water layer and were accompanied by pronounced seasonality with an interannual variation of 5.2°C in La Quebrada and 5.5°C in El Aerolito. The mesohaline layer from El Aerolito followed the same pattern, except 2°C colder. Caves without direct connections to the sea showed a smaller interannual variation, 0.5°C in Tres Potrillos and 0.7°C in Bambú.

In contrast, water temperature in La Quebrada, at the farthest point from the coastline (site “c”, Fig 1), had an interannual variation of only 0.7°C (Fig 2). This was similar to caves without direct connections to the sea. The morphology of the cave can create a “two caves” system, in terms of temperature stability. Although mesohaline and mixoeuhaline water layers are present in both sections, a blockage of some type prevents direct communication. As a result, the seaward section of the cave shows seasonable temperature variation, while the isolated part has a stable temperature.

Precipitation was recorded continuously between January 2015 and July 2016. These records include storms through the year (maximum precipitation was 133mm) and any hurricane that impacted Cozumel Island. Corresponding to the biggest storms, the cave water temperature dropped drastically in the same magnitude as the interannual variation (≈5°C) in La Quebrada, El Aerolito and Tres Potrillos (≈0.5°C). In contrast, the temperature in Bambú increased on only one occasion (0.4°C). During the temperature measurements, eight severe temperature drops were identified, all of them corresponding to storms with rainfall of at least 30mm (Fig 2, S2 Table).

Interestingly, in El Aerolito the day after an eight-day storm (October 13–20, 2015), a strong current was flowing in the direction to the sea. In addition, a mixing zone of the mesohaline and mixoeuhaline water, as opposed to a marked halocline, was at 12m in depth (5m deeper than usual). As a consequence, mobile benthic organisms moved to deeper zones. In particular, the ophiuroid *Ophionereis* sp. (the most abundant species in that cave) aggregated in large numbers, one above the other. In areas above the mixing zone (12m depth), a large number of dead, or very weak (evident by low reaction to direct light and physical stimuli) organisms of most of the species (i.e. ophiuroids, ascidians, polychaetes) were found (S1 Video). Unfortunately, it was not possible to made post-storms observations in other caves.

### Biological surveys

**Fig 3:**
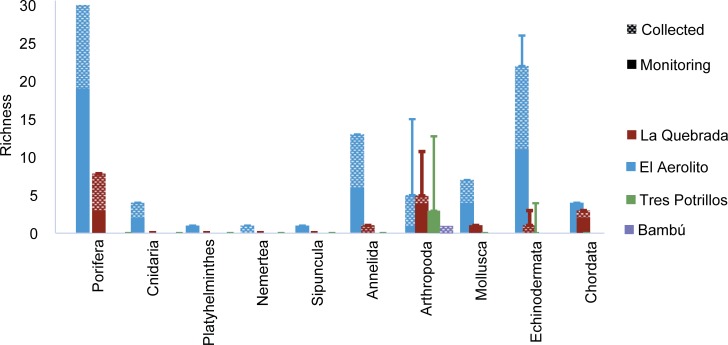
Species richness of the anchialine caves. Error bar refers to species recorded in the literature, but not observed in this study. Monitoring refers to data collected through visual census.

A total of 795m^2^ was surveyed. Surveys were distributed in four sites in El Aerolito, three in La Quebrada, two in Tres Potrillos and one in Bambú. Each survey site had 2 to 12 replicates, for a total of 54 surveys (Fig 1). Macrofauna biodiversity was dependent on the nature of the connection with the sea. A total of 97 OTUs were identified: 87 OTUs from 10 phyla in El Aerolito and 19 OTUs of 6 phyla in La Quebrada. On the other hand, only three species of crustaceans were found in Tres Potrillos and one in Bambú (Fig 3). The asymptote of the species accumulation curve was reached in each cave (S1 Fig). The species composition was significantly different with only five species occurring in more than one cave (S3 Table).

During the biological census, 55 OTUs were observed: 10 OTUs in La Quebrada, 47 OTUs in El Aerolito, and 3 OTUs in Tres Potrillos. In contrast, no species were found in Bambú (only two organisms, that were not identified, were observed during the fieldwork, neither inside a transect) (Fig 3). Most species show a restricted distribution inside caves (Table 1; complete species richness including bibliographical records and population estimation size is available in the supplemental material S3 Table).

**Table 1:** Distribution of the OTUs recorded during the biological census per anchialine cave. The order of the taxa is in accordance with the distribution range of each OTU. A list of all the species is available in S3 Table.

		Site			
Cave	Taxa	a	b	c	d
El Aerolito	*Ascidia* sp. 1	1	1	1	1
	Calcarea sp. 2	1	1	1	1
	*Gastrophanella* sp. *& Aciculites higginsii*	1	1	1	1
	*Geodia neptuni*	1	1	1	1
	*Notopygos caribea*	1	1	1	1
	*Ofionereis reticulata*	1	1	1	1
	*Polychaeta* sp.	1	1	1	1
	*Pyura* cf. *munita*	1	1	1	1
	*Dorvillea moniloceras*	1	1	1	
	*Volvarina avena*	1	1	1	
	*Acarnus innominatus*	1	1		1
	Agelasidae	1	1		1
	*Geodia* sp. *2*	1	1		1
	*Ophiomusa* cf. *testudo*	1	1		
	Actiniaria	1			
	Calcarea sp. 4	1			
	*Cypraea zebra*	1			
	*Eucidaris tribuloides*	1			
	*Haliclona (Reniera)* sp. 1	1			
	*Plakortis angulospiculatus*	1			
	*Diplastrella megastellata*		1	1	1
	*Geodia* sp. 1		1	1	1
	*Placospongia* sp.		1	1	1
	*Tethya* sp. 1 & sp. 2		1	1	1
	*Ophiothrix (Ophiothrix) oerstedii*		1	1	
	Sipuncula		1	1	
	*Ophiothrix (Ophiothrix) angulata*		1		1
	*Ascidia* sp. 2		1		
	*Balanophyllia (Balanophyllia) bayeri*		1		
	Chondrosiida		1		
	*Ctenoides scaber*		1		
	*Harmothoe* sp.		1		
	*Hermodice carunculata*		1		
	*Mithrodia clavigera*		1		
	*Plakortis* sp.		1		
	*Polychaeta* sp. 2		1		
	Turbellaria		1		
	*Typhliasina pearsei*		1		
	*Penaeus* sp.			1	1
	*Copidaster cavernicola*			1	
	*Holothuria (Semperothuria) surinamensis*			1	
	*Ophiocoma wendtii*			1	
	*Ophiothrix (Acanthophiothrix) suensonii*			1	
	*Asterinides* sp.				1
	Heterobranchia				1
	*Stelletta* sp.				1
La Quebrada	*Didemnum* sp.	1	1	1	
	*Diplastrella* sp.	1		1	
	*Haliclona (Reniera)* sp. 2	1		1	
	*Mayaweckelia* sp.	1		1	
	*Discodermia adhaerens*	1			
	*Typhliasina pearsei*	1			
	*Metacirolana mayana*		1	1	
	*Bahadzia bozanici*		1		
	*Procaris mexicana*		1		
	*Cyclostrema cancellatum*			1	
Tres Potrillos	*Mayaweckelia* sp.	1	1		
	*Metacirolana mayana*	1	1		
	*Procaris mexicana*	1	1		

During the field work *Gastrophanella* sp. & *Aciculites higginsi* were identified as *Gastrophanella* sp.; and *Tethya* sp. 1 & sp. 2 were identified as *Tethya* spp.

**Fig 4:**
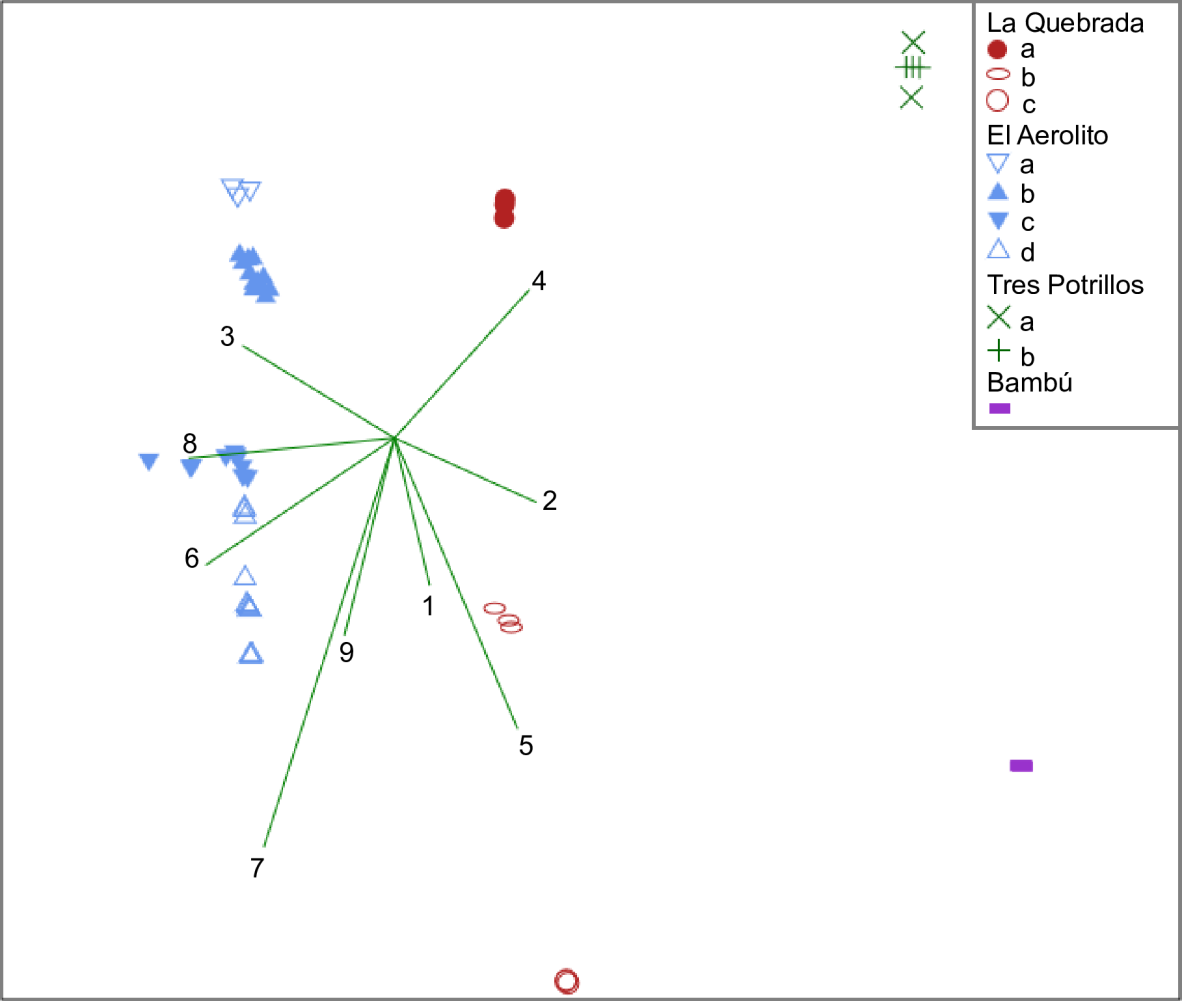
Non-metric multidimensional analysis (nMDS, stress = 0.065, R^2^ axis 1 = 0.970, R^2^ axis 2 = 0.131). Lines show the significance of abiotic parameters: 1) substratum type, 2) depth, 3) temperature, 4) organic matter in the sediment (%), 5) distance of the site to the sea, 6) type of connection to the sea, 7) distance to the nearest entrance, 8) cave length, 9) distance to the halocline. Each symbol represents a survey.

**Fig 5:**
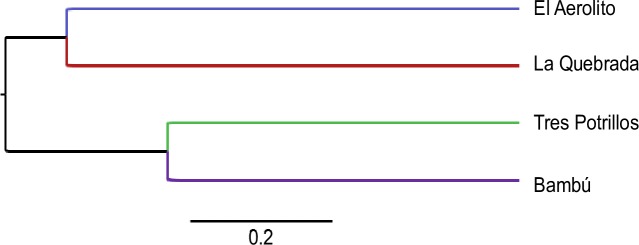
Similitude analysis (Bray-Curtis index). The analysis was performed based on density data of the recorded fauna.

The nMDS analysis (Fig 4) strongly supports the distinction between each cave, separating them without overlap (stress = 0.065, R^2^ axis 1 = 0.970, R^2^ axis 2 = 0.131). This separation was related to the type of connection with the sea, length, and depth of the cave. Separation between regions inside of each anchialine cave was also supported. The abiotic factors that support the regionalization within the caves are the distance to the nearest entrance, distance to the sea, distance of the halocline to the bottom of the cave, and the substratum type. The ordination analysis (Fig 5) shows low similarity, but the caves were grouped in accordance to their type of connection with the sea. Therefore, both analyses support the premise that each anchialine cave has a unique community structure and that species richness is dependent on the nature of connection with the sea.

Biological census data from El Aerolito and La Quebrada were not normal (S4 Table) nor homoscedastic (El Aerolito: df = 3, p < 0.05; La Quebrada: df = 2 p < 0.05). The specific average density per site was 0.004 to 11.534org/m^2^ with a mean of 0.046org/m^2^. Only seven OTUs had densities of 0.5org/m^2^, five of them in El Aerolito. This data suggests that the fauna is mostly characterized by low density. In all cases, high standard deviation values found present because of the low distribution uniformity of organisms (S5 Table). Due to its relatively small size, Tres Potrillos only had two census sites. Therefore, it was not possible to perform statistical analyses.

### Taxonomic composition

In La Quebrada, a distinctive taxonomic composition was observed among sites (Fig 3). However, these differences were not significant (K-W = [2, N = 36]) = 3.977, p > 0.05). The box plot (S2 Fig) shows that the highest density was recorded at site “a”, which is the closest to the sea.

The densities as well as the distribution per phyla in El Aerolito were similar at sites a-c, but significantly different from site “d” (K-W [3, N = 333] = 24.62, p < 0.05). *A posteriori* test confirmed that site “d” was significantly different from sites “b” and “c”, while the difference between sites “a” and “d” was not significant (S4 Table). In the box plot, sites a-c from El Aerolito matched, but differed from site “d”. The box plot results correspond to the field observations (S2 Fig).

Only crustaceans were recorded in Tres Potrillos, with the deeper site having a nine-fold greater density (S2 Fig).

### Population size estimation

The estimate of population size of all species recorded in the biological census was obtained by extrapolation of their densities. Divisions of each anchialine cave were based on the results of the nMDS, Kruskal-Wallis and field observations. The population size was estimated by clustering La Quebrada sites “a” and “b”, resulting in two regions; El Aerolito sites “b” and “c”, resulting in three regions; while Tres Potrillos sites were not clustered.

The estimate of total population size (S3 Table) yielded a total of 577,994 (SD = 442,983) organisms in the four anchialine caves. El Aerolito accounted for 82.0% of the organisms (437,731, SD = 396,301), followed by La Quebrada with the 17.8% (103,017, SD = 46,443), and Tres Potrillos with only 0.2% (1,245, SD = 240). The extremely low density and the limited number of sites surveyed in Bambú did not allow for an estimation of the population size. La Quebrada had the largest mean density per species (10,302, SD = 21,588), while El Aerolito had a smaller value, but higher dispersion (9,475, SD = 38,213) due to the dominance of the ophiuroid *Ophionereis* sp. The lowest abundance per species was in Tres Potrillos (415, SD = 509). *Ophionereis* sp. was the most abundant species with 268,365 (SD = 131,735) individuals, while *Ophicoma wendtii* was the rarest with 36 individuals (SD = 116); both extreme values were recorded in El Aerolito (S3 Table).

## Discussion

### Biodiversity

Underwater caves present a distinct series of challenges and therefore require a high degree of specialization by the organisms inhabiting them [4]. At the same time, they are semi-isolated ecosystems comparable with islands [8]. As a result, the community structure in the dark zone of each cave can be unique, and the population sizes can be very low [3,20,26,27].

Besides the 97 OTUs that we observed, 25 other species were previously reported in the same caves (Fig 3), resulting in a total of 122 species records: 101 in El Aerolito, 27 in La Quebrada and seven in Tres Potrillos (S3 Table) [5,19,30,46,47,53–55]. In general, the species that were not observed during this study were either reported near cenote entrances [56,57], collected using plankton nets because of their small size [58,59], or were species that usually live in open water that had been reported inside of the cave only once (stygoxene or accidental fauna) [18,60,61]. Nevertheless, the crustaceans *Metacirolana mayana* (in El Aerolito), *Parhippolyte sterreri*, or *Yagerocaris cozumel*, were not observed, although they are stygobitic species reported multiple times [24,46,58,62]. Neither was observed any remipedes in El Aerolito, which were only observed once in 2005 by local cave divers. This suggests that their populations are likely rare.

Only 10 species were observed in more than one cave (S3 Table): El Aerolito and La Quebrada shared the amphipod *Bahadzia bozanici;* the shrimps *Yagerocaris cozumel* and *Agostocaris zabaletai;* the marine sponges *Discodermia adhaerens* and *Plakinastrella onkodes;* the sea star *Asterinides* sp., and the irregular sea urchin *Brissopsis atlantica*. La Quebrada and Tres Potrillos shared the amphipod *Mayaweckelia* sp. and the isopod *Metacirolana mayana*. The shrimp *Procaris mexicana* was found in El Aerolito, La Quebrada and Tres Potrillos, but not in Bambú [5,20,47,54,55]. This indicates that the specific composition of each anchialine cave is unique, a pattern previously observed for sponges and fishes in Mediterranean marine caves [3,26].

### Ecological patterns

**Fig 6:**
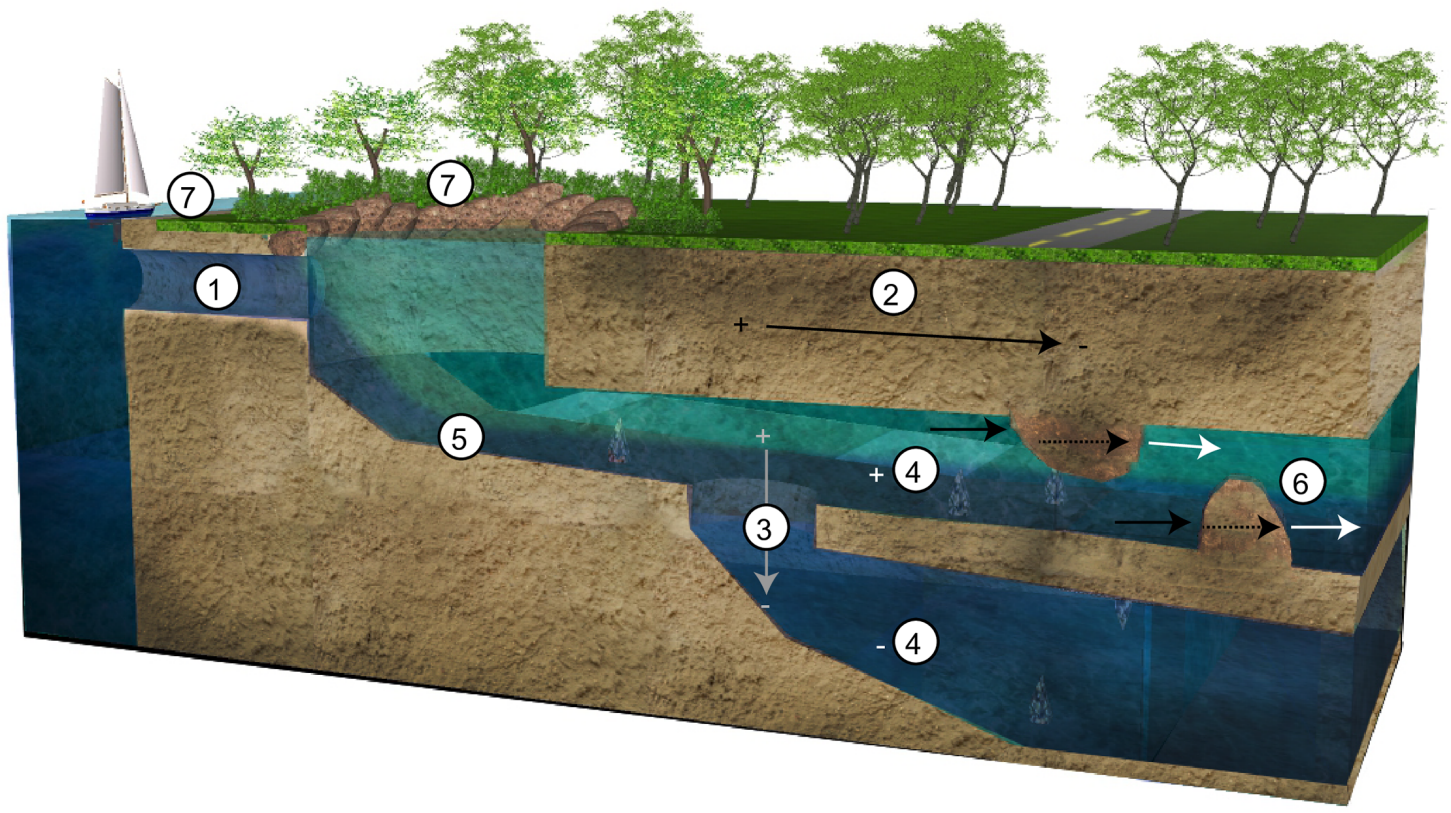
Diagram explaining the ecological patterns concerning the species richness, taxonomic composition and expected density. 1) Increase in caves with direct connection to the sea. 2) Decrease as the distance to the sea increases. 3) Decrease bathymetrically. 4) Decrease in areas without halocline. 5) Decrease in areas where the halocline is close to the bottom. 6) Species richness and diversity can dramatically change in caves due to discontinuities, which generate different community structures. 7) Adjoining ecosystems determine the available quantity and quality of organic matter in the cave.

By analyzing the species richness, taxonomic composition and density of conspicuous fauna, with reference to geomorphological and abiotic attributes of the caves, seven ecological patterns emerged (Fig 6):

Type of connection with the sea **(1)**. Caves with a direct connection to the sea have a greater richness and diversity (Fig 6.1). The direct connection allows the influx of organisms, including larvae stages, increasing richness by the combination of stygobitic, stygophile and stygoxene fauna [19,60,63]. This pattern could also explain the higher richness in marine caves in comparison with anchialine caves [3,26]. Additionally, the direct connection with the sea facilitates entry of important energy sources including dissolved and particulate organic matter and plankton [64–66].

Distance from the sea **(2)**. Species richness diminished as the distance from the sea (or coastline) increased, even within the same cave (Fig 6.2). As the linear distance into a cave increases, the quantity of available organic matter is reduced [67], decreasing both species richness and density. This pattern was shown in all areas, except those with chemosynthesis, such the site “c” in El Aerolito, where chemosynthetic production of dissolved organic carbon allows a greater density of macroorganisms [33,65]. In fact, the highest species richness in terrestrial and underwater caves has been reported near their entrances [3,4,26,68]. Caves lacking a direct connection to the sea, but still being close to the coastline, exhibit an increased probability that “new” organisms can be transported into the cave as a result of hydrological changes, such as sea level rise [42,69]. Therefore, species richness decreased in caves, inland and farther from the coastline. However, stygobitic species are present in a greater proportion in caves farther from the coastline, where the introduction of new organisms is less feasible. This also explains why the greatest species richness is known in caves close to the coastline [20] and why higher species richness is found in marine caves in comparison with anchialine caves [3,26].

Water depth **(3)**. Species richness decreased as the water depth increased, even within the same cave (Fig 6.3). This is a consequence of less organic matter being carried into deeper parts of the cave, analogous to the deep sea [70]. The reduction of richness and density occurs with both horizontal distance and water depth.

Presence of halocline **(4)**. The presence of a halocline dramatically increased species richness (Fig 6.4). Due to the geomorphology of some anchialine caves, not all sections have a halocline, especially in passages with low floor to ceiling heights. This causes a drastic decrease in species richness and density because animals do not have access to multiple water layers. The halocline works as a “conveyor belt” of organic matter from the surface environments. Also, the presence of the halocline means multiple water layers and multiple environments [44,71]. Low richness and densities at site “d” of El Aerolito could be explained in this manner.

Halocline depth **(5)**. Species richness decreased in areas where the halocline is located close to the bottom. In the event of an increase in halocline depth (e.g. by tide, storms), the fauna would be exposed to abrupt thermal and osmotic stress (Fig 6.5). Anchialine caves are under tidal influence such that the halocline depth changes are in accordance with the tide and distance to the coastline [1]. For example, in Edén, an anchialine cave on the mainland of the Yucatan Peninsula near Puerto Aventuras, Quintana Roo, a 2m variation of the halocline’s depth occurs as a result of the tides [72]. In comparison, a -5m variation in El Aerolito was observed as a consequence of strong storms. This could explain the low richness of La Quebrada in comparison with El Aerolito, even when both share many characteristics [20,46,48]. The lower depth of La Quebrada results in a halocline close to the cave floor, so it is reasonable to assume that organisms are exposed to recurring osmotic and thermal stress, making those areas only habitable to eurythermal and euryhaline species [11,73].

Continuity of the cave, or presence of barriers inside **(6)**. Species richness and diversity dramatically changed in caves due to discontinuities (Fig 6.6). This could be due to dry zones [74] or because a restriction to a single water layer, resulting from the cave’s morphology, even if both layers are present in the next section (as in site “c” of La Quebrada). Although divers can swim through different water masses, physical barriers can isolate water masses of varying salinity as has been observed in estuaries [11]. Such barriers result in the presence of different community structures in sections of the same cave.

Adjoining ecosystems **(7)**. Ecosystems interacting with the anchialine ecosystem, particularly at their entrances, determine the quantity and quality of organic matter [44,65], as well as the fauna entering to the cave (Fig 6.7). Greater species richness and density would be expected in caves that are connected with energy exporting ecosystems, such as mangroves [75], as well as in those caves with multiple connections to the sea or to the land surface.

### Potential effects of climate change and conservation implications

Caves, either on land or underwater, are relatively stable environments, with small, mostly predicable fluctuations. For underwater caves, this stability is dependent on the morphology of the cave and its interaction with adjoining ecosystems [4,76]. Medium-term temperature changes (such as global warming) can impact the fauna in subaquatic caves in important ways (e.g., dramatic mass mortalities were reported in Mediterranean caves) [17,77]. However, until now, the high impact of single events such as storms or hurricanes has never been reported. Considering climate change models project an increase in frequency and strength of hurricanes and other severe storms, the risk for species inhabiting shallow anchialine caves with haloclines close the bottom is magnified [78,79].

Most of the phyla recorded in this research have been extensively studied in the adjacent marine area [80–86]. The crustaceans *Barbouria yanezi*, and *Stenobermuda* sp., the sea stars *Copidaster cavernicola*, and *Asterinides* sp., the ophiuroid *Ophionereis* sp., the marine sponges Agelasidae, *Dercitus* sp., *Gastrophanella* sp., *Haliclona (Reniera)* sp. 1, *Haliclona (Reniera)* sp. 2, *Leiodermatium* sp., *Lytechinus* sp., *Microscleroderma* sp., and *Psammastra* sp. (species at genus level are currently under description) could be potential micro-endemic species (our study area covers approximately a total of 0.09km^2^). From the recorded species in the biological census, the sea stars *Asterinides* sp. and *C*. *cavernicola*, as well as the marine sponge *Gastrophanella* sp., had an estimated population size of less than 1,800 individuals. It is important to notice that the population size of mature organisms may be significantly lower since it was not possible to make this distinction. This value is under the 2,500 individuals within 10km^2^ criteria of the IUCN to recognize a species under extinction danger (criteria B2, C, D), or critically endangered (criteria B2) [87]. Therefore most, if not all, endemic anchialine species could already be under extinction risk, especially considering the strong anthropogenic pressure in the area (e.g., deforestation, coastal development, deep well injection) [36,39,88]. This reinforces the conclusion of the high extinction risk anchialine cave fauna now faces and strongly supports the implementation of *ad hoc* conservation strategies for each cave [3,7,27,32].

## Supporting information

S1 Video Current inside of El Aerolito and the behavior of its fauna after a strong storm. (RAR)

S1 Fig Accumulation species’ curve. (DOCX)

S2 Fig Density of macro-organisms per surveyed site. (DOCX)

S1 Table Abiotic characteristics of the censed sites. (DOCX)

S2 Table Temperature and precipitation. (PDF)

S3 Table Population estimation size (value) and presence (*) of macrofauna in the anchialine caves of Cozumel. Stygobitic species (^+^).
(DOCX)

S4 Table *A priori* and *a posteriori* statistics. (DOCX)

S5 Table Specific density (org/m^2^) per censed site with its standard deviation (SD). (DOCX)
