# Supplementary material for: Ecological patterns in anchialine caves
Source: PLoS One. 2018 Nov 7;13(11):e0202909. doi: 10.1371/journal.pone.0202909 (PMC6221257; doi:10.1371/journal.pone.0202909)
Supplement: S1 Fig — (DOCX) [file pone.0202909.s002.docx]

**S1 Figure. –** **Richness vs effort.** Species accumulation curves. During sampling (green – dive number), and rarefaction curve with the census data (red and its standard deviation in blue - transects). A) El Aerolito, B) La Quebrada and, C) Tres Potrillos.


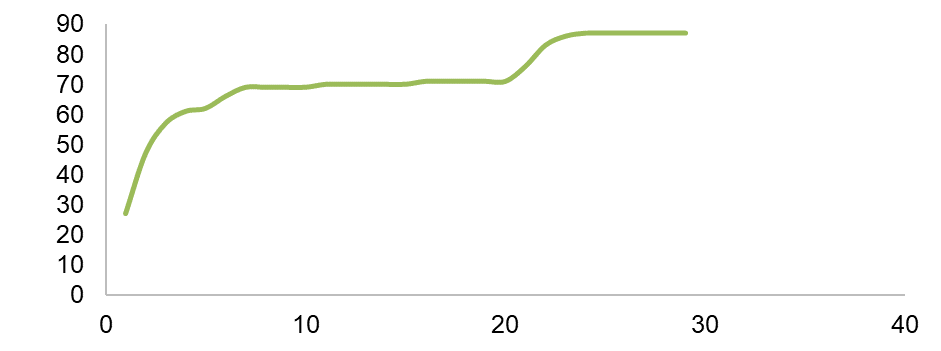

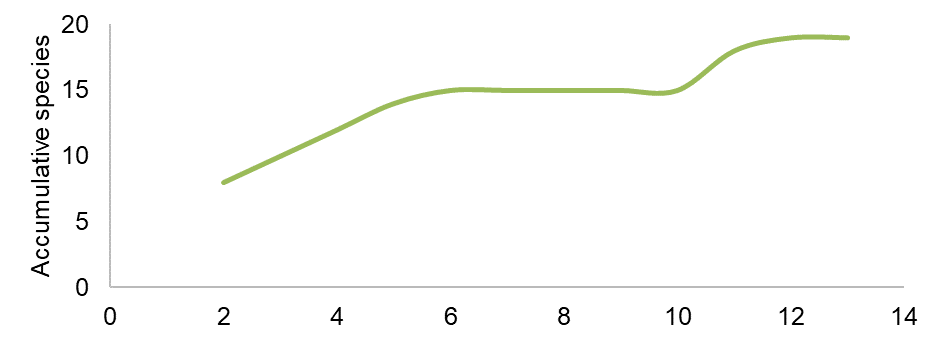

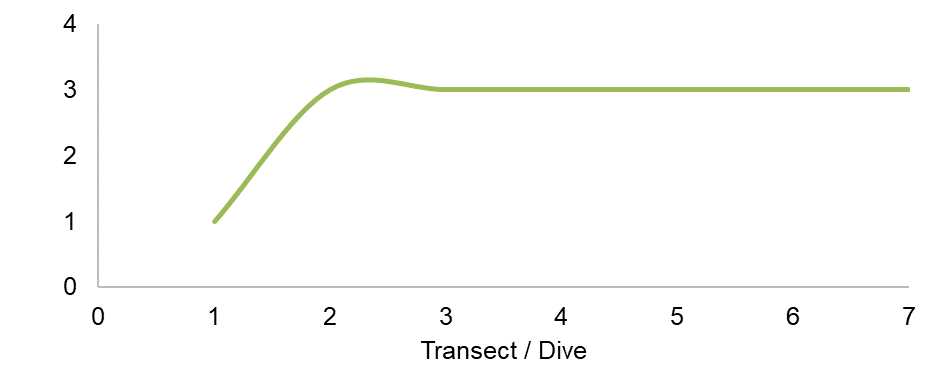

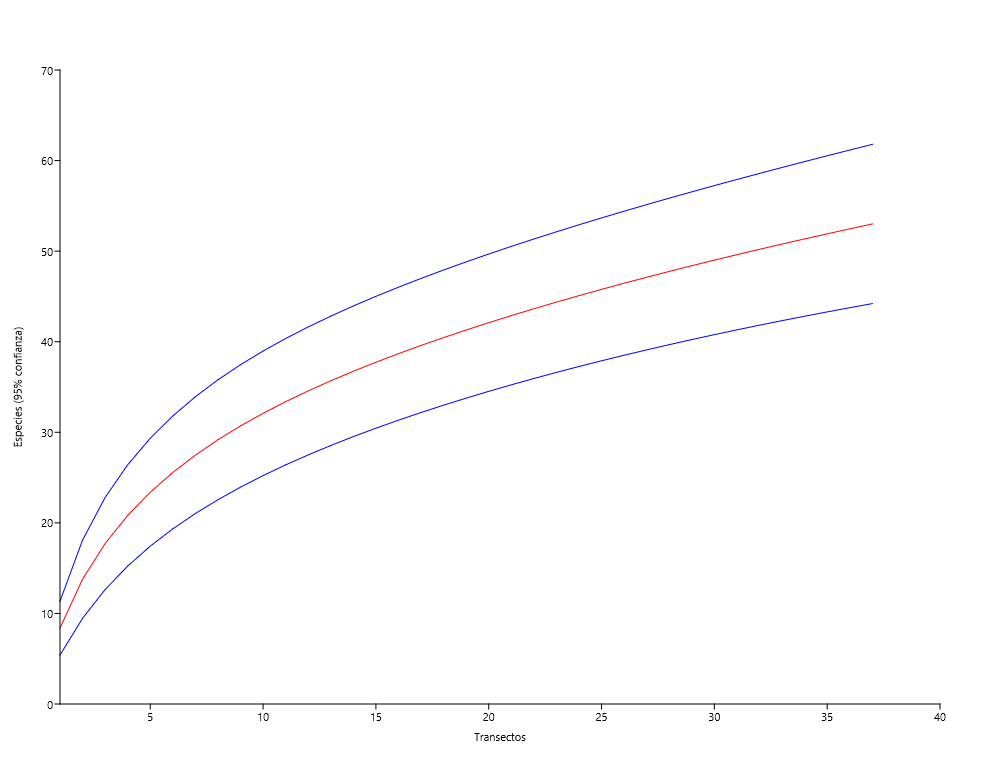

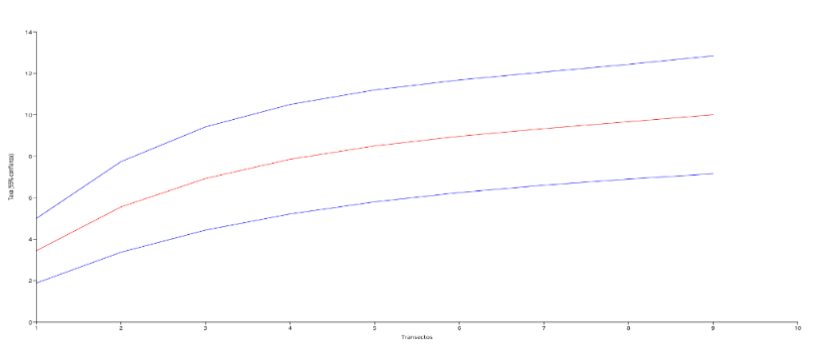

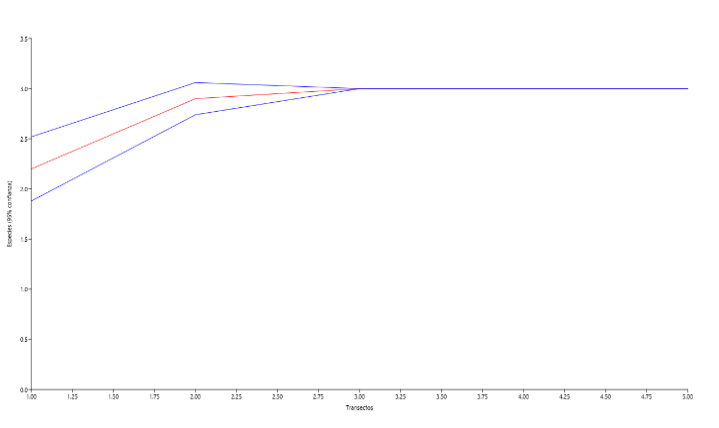


a

b

c
