# Supplementary material for: Ecological patterns in anchialine caves
Source: PLoS One. 2018 Nov 7;13(11):e0202909. doi: 10.1371/journal.pone.0202909 (PMC6221257; doi:10.1371/journal.pone.0202909)

**S2 Figure. –** Density of macro-organisms per surveyed site. Standard deviation is shown with error bars.


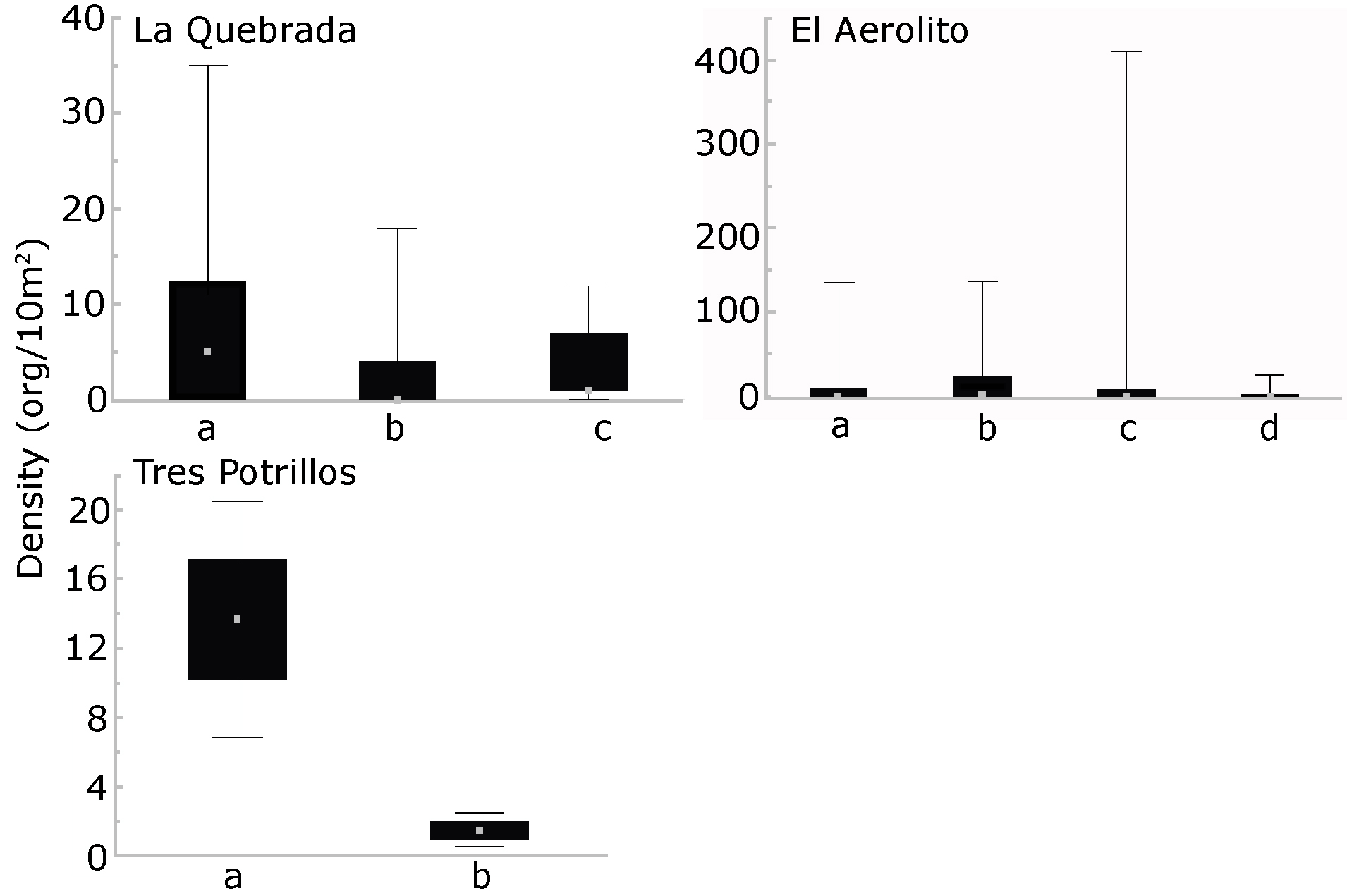

Supplement: S2 Fig — (DOCX) [file pone.0202909.s003.docx]
