# Supplementary material for: Ecological patterns in anchialine caves
Source: PLoS One. 2018 Nov 7;13(11):e0202909. doi: 10.1371/journal.pone.0202909 (PMC6221257; doi:10.1371/journal.pone.0202909)
Supplement: S1 Table — (DOCX) [file pone.0202909.s004.docx]

**S1 Table. - Abiotic characteristics of the surveyed sites.** Substratum of the cave bottom: 1) principally mud-clay, 2) principally rock, 1/2) transects with either rock and/or mud-clay. Mean values are given for depth, temperature (Temp.) and distance to the halocline (Dist. Halocline). Percentage of organic matter (% O.M.). Connection to the sea: 1) direct, 0) indirect. Distance to the closest exit (Dist. exit.).

| Site | Substratum | Depth (m) | Temp. (°C) | % O.M. | Dist. to sea (m) | Connection with sea | Dist. exit (m) | Length (m) | Dist. Halocline (m) |
| --- | --- | --- | --- | --- | --- | --- | --- | --- | --- |
| El Aerolito |  |  |  |  |  |  |  |  |  |
| a | 1 | 11 | 28 | 2.867 | 225 | 1 | 187.5 | 18,000 | 4 |
| b | 1 | 9 | 27 | 1.974 | 285 | 1 | 140 | 18,000 | 2 |
| c | 1 | 10 | 28 | 2.574 | 379 | 1 | 292 | 18,000 | 3 |
| d | 1 | 18 | 27 | 3.735 | 418 | 1 | 297 | 18,000 | 100 |
| La Quebrada |  |  |  |  |  |  |  |  |  |
| a | 1/2 | 4.8 | 27.8 | 2.356 | 216 | 1 | 126 | 9,226 | 0.8 |
| b | 1/2 | 7.5 | 26.6 | -0.322 | 526 | 1 | 155 | 9,226 | 3.5 |
| c | 1/2 | 6 | 27.8 | 1.463 | 833 | 1 | 402 | 9,226 | 2 |
| Tres Potrillos | | |  |  |  |  |  |  |  |
| a | 1 | 36.1 | 26 | 56.017 | 268 | 0 | 20 | 62 | 25.75 |
| b | 1 | 16.3 | 26 | 0.000 | 268 | 0 | 20 | 62 | 5.95 |
| Bambú |  |  |  |  |  |  |  |  |  |
|  | 1 | 45 | 22 | 23.559 | 5263 | 0 | 30 | 60 | 3 |
