# Supplementary material for: Ecological patterns in anchialine caves
Source: PLoS One. 2018 Nov 7;13(11):e0202909. doi: 10.1371/journal.pone.0202909 (PMC6221257; doi:10.1371/journal.pone.0202909)
Supplement: S2 Table — (PDF) [file pone.0202909.s005.pdf]

S2 Table. Temperature and precipitation

Date	Temperature								Precipitation
	El Aerolito				La Quebrada		Tres Potrillos	Bambú	
	Entrance	At 100m from entrance	Site "c"	Site "d"	Site "a"	Site "c"			
07/01/2015			27.20						0
08/01/2015	26.80		27.22						0
09/01/2015	26.86		26.97						0
10/01/2015	26.79		26.95						0
11/01/2015	26.89		26.94						0
12/01/2015	27.00		27.10		27.18				0
13/01/2015	27.02		27.08		27.19				0
14/01/2015	27.06		27.13		27.17				0
15/01/2015	27.05		27.05		27.06				0
16/01/2015	27.07		27.10		27.09				0
17/01/2015	27.01		27.10		27.06				0
18/01/2015	27.07	25.31	27.12	27.32	27.04				0
19/01/2015	27.04	25.29	27.18	27.35	27.06		26.00		0
20/01/2015	27.03	25.30	27.13	27.33	27.07		26.00		0
21/01/2015	27.07	25.32	27.20	27.33	27.11		26.00		0
22/01/2015	27.10	25.35	27.17	27.18	27.10		26.00		0
23/01/2015	27.23	25.36	27.24	27.28	27.18		26.00	21.92	0
24/01/2015	27.19	25.36	27.29	27.42	27.25		26.00	21.94	0
25/01/2015	26.82	25.27	26.98	27.10	27.02		26.00	21.91	0
26/01/2015	26.77	25.23	26.99	26.96	27.00		26.00	21.91	0
27/01/2015	26.64	25.21	26.94	27.11	27.08		26.00	21.92	0
28/01/2015	26.67	25.18	26.73	26.76	26.91		26.00	21.89	0
29/01/2015	26.81	25.21	26.86	26.79	26.98		26.00	21.91	0
30/01/2015	26.83	25.21	26.92	26.85	27.03		26.00	21.93	0
31/01/2015	26.74	25.20	26.88	26.81	27.03		26.00	21.91	0
01/02/2015	26.87	25.22	26.91	26.91	27.03		26.00	21.91	0
02/02/2015	26.91	25.24	27.00	26.99	27.07		26.00	21.91	0
03/02/2015	26.89	25.24	27.00	27.02	27.10		26.00	21.91	0
04/02/2015	26.91	25.24	27.06	27.08	27.13		26.00	21.90	0
05/02/2015	26.89	25.25	27.05	27.01	27.12		26.00	21.90	0
06/02/2015	26.58	25.21	27.03	27.15	27.17		25.99	21.90	0
07/02/2015	26.47	25.13	26.56	26.64	26.92		26.00	21.89	0
08/02/2015	26.72	25.15	26.82	26.77	27.02		26.00	21.92	0
09/02/2015	26.80	25.21	26.93	26.90	27.05		26.00	21.88	0
10/02/2015	26.91	25.22	26.97	26.95	27.07		26.00	21.89	0
11/02/2015	26.71	25.22	26.84	26.92	26.92		26.00	21.88	0
12/02/2015	26.95	25.20	26.94	26.91	26.93		26.00	21.90	0
13/02/2015	26.69	25.21	26.93	26.85	26.97		26.00	21.91	0
14/02/2015	26.64	25.17	26.80	26.85	26.92		26.00	21.90	0
15/02/2015	26.50	25.16	26.74	26.94	26.89		26.00	21.90	0
16/02/2015	26.52	25.17	26.69	26.61	26.80		26.00	21.91	0
17/02/2015	26.55	25.16	26.69	26.61	26.79		26.00	21.91	0
18/02/2015	26.56	25.17	26.75	26.78	26.80		26.00	21.91	0
19/02/2015	26.02	25.10	26.62	26.88	26.68		26.00	21.90	0
20/02/2015	26.01	25.02	26.34	26.20	26.46		26.00	21.91	0

21/02/2015	26.24	25.06	26.41	26.35	26.65	26.00	21.90	0
22/02/2015	26.45	25.10	26.54	26.51	26.76	26.00	21.90	0
23/02/2015	26.56	25.11	26.62	26.66	26.87	26.00	21.89	0
24/02/2015	26.70	25.15	26.64	26.67	26.88	26.01	21.88	0
25/02/2015	26.79	25.22	26.77	26.79	26.92	26.05	21.87	0
26/02/2015	26.78	25.23	26.76	26.73	26.92	26.09	21.88	0
27/02/2015	26.87	25.21	26.85	26.85	26.92	26.08	21.88	0.77
28/02/2015	26.86	25.23	26.84	26.87	26.99	26.08	21.89	0
01/03/2015	26.93	25.25	26.89	26.91	26.99	26.09	21.88	0
02/03/2015	26.88	25.24	26.93	26.96	27.01	26.08	21.87	0.25
03/03/2015	26.86	25.25	26.89	26.90	26.98	26.09	21.87	0
04/03/2015	26.91	25.25	26.92	26.98	27.01	26.10	21.88	0
05/03/2015	26.83	25.23	26.94	26.92	26.99	26.10	21.88	0
06/03/2015	26.93	25.21	26.94	26.98	27.01	26.10	21.87	0
07/03/2015	26.90	25.21	26.94	27.07	27.05	26.09	21.88	0
08/03/2015	26.89	25.24	26.97	26.97	27.01	26.10	21.87	0
09/03/2015	26.96	25.23	26.99	27.02	27.06	26.10	21.86	0
10/03/2015	26.95	25.24	26.99	27.00	27.07	26.09	21.86	0
11/03/2015	27.00	25.22	26.94	26.98	27.11	26.08	21.86	0.25
12/03/2015	27.04	25.23	26.97	27.00	27.09	26.08	21.88	0
13/03/2015	27.14	25.26	27.00	27.09	27.11	26.08	21.87	0
14/03/2015	27.15	25.29	27.08	27.10	27.10	26.08	21.88	0
15/03/2015	27.13	25.27	27.07	27.09	27.14	26.07	21.89	0
16/03/2015	27.13	25.27	27.06	27.12	27.12	26.05	21.90	0
17/03/2015	27.07	25.29	27.08	27.12	27.09	26.05	21.90	0
18/03/2015	27.10	25.30	27.08	27.14	27.07	26.03	21.89	0
19/03/2015	27.10	25.32	27.12	27.19	27.11	26.01	21.89	0
20/03/2015	27.08	25.33	27.11	27.23	27.11	26.00	21.89	0
21/03/2015	27.03	25.30	27.10	27.26	27.06	26.00	21.90	0
22/03/2015	27.04	25.31	27.10	27.21	27.09	26.00	21.88	0
23/03/2015	27.08	25.32	27.12	27.15	27.17	26.00	21.88	0.26
24/03/2015	27.13	25.32	27.18	27.27	27.24	26.00	21.87	0
25/03/2015	27.15	25.28	27.15	27.21	27.23	26.00	21.87	0
26/03/2015	27.32	25.30	27.18	27.20	27.27	26.00	21.86	2.29
27/03/2015	27.30	25.38	27.28	27.30	27.29	26.00	21.87	33.78
28/03/2015	27.08	25.28	27.19	27.34	27.26	26.00	21.88	2.79
29/03/2015	26.86	25.26	27.03	27.34	27.16	26.00	21.86	0
30/03/2015	26.93	25.26	27.03	27.18	27.15	26.00	21.87	1.02
31/03/2015	26.91	25.25	27.03	27.26	27.09	26.00	21.86	0
01/04/2015	26.93	25.26	27.03	27.19	27.08	26.00	21.86	0
02/04/2015	26.93	25.23	27.07	27.36	27.07	26.00	21.86	0
03/04/2015	26.78	25.23	27.01	27.27	27.04	26.00	21.87	0
04/04/2015	26.67	25.11	26.77	27.12	27.04	26.00	21.87	0
05/04/2015	26.62	25.12	26.78	27.16	27.04	26.00	21.88	0
06/04/2015	26.88	25.14	26.83	27.18	27.04	26.00	21.86	0
07/04/2015	26.38	25.07	26.59	27.01	27.00	26.00	21.92	0
08/04/2015	26.45	25.00	26.46	26.92	27.20	26.00	21.86	0
09/04/2015	26.26	24.96	26.37	26.88	27.17	26.00	21.87	0
10/04/2015	26.30	24.96	26.32	26.85	27.24	26.00	21.86	0
11/04/2015	26.16	24.92	26.15	26.79	27.12	26.00	21.86	0

12/04/2015	26.15	24.87	26.02	26.71	27.19	25.99	21.87	0
13/04/2015	26.39	24.92	26.21	26.73	27.14	26.00	21.87	0
14/04/2015	26.29	24.91	26.11	26.73	27.03	26.00	21.88	0
15/04/2015	26.34	24.91	26.18	26.77	26.94	26.00	21.88	0
16/04/2015	26.34	24.89	26.16	26.77	26.99	26.00	21.88	0
17/04/2015	26.77	25.03	26.49	26.90	26.98	26.00	21.87	0
18/04/2015	27.07	25.20	26.90	27.13	27.02	26.00	21.88	0
19/04/2015	26.99	25.18	26.85	27.08	27.04	26.00	21.88	0
20/04/2015	26.80	25.11	26.67	27.11	27.00	26.00	21.87	0
21/04/2015	27.01	25.14	26.77	27.14	27.07	26.00	21.86	0
22/04/2015	27.12	25.20	26.86	27.15	27.17	26.00	21.86	0
23/04/2015	26.47	24.97	26.32	26.92	27.19	26.00	21.86	0
24/04/2015	26.95	25.11	26.70	27.06	27.15	26.00	21.86	0
25/04/2015	27.47	25.24	27.20	27.21	27.28	26.00	21.86	0
26/04/2015	27.83	25.55	27.61	27.68	27.44	26.03	21.86	0
27/04/2015	27.57	25.40	27.46	27.57	27.38	26.01	21.86	0
28/04/2015	27.41	25.39	27.37	27.47	27.35	26.02	21.86	0.25
29/04/2015	27.82	25.57	27.68	27.74	27.55	26.08	21.86	0.25
30/04/2015	27.35	25.42	27.47	27.53	27.44	26.06	21.87	1.27
01/05/2015	27.24	25.36	27.22	27.28	27.33	26.09	21.86	0.26
02/05/2015	27.32	25.35	27.28	27.44	27.33	26.07	21.87	14.73
03/05/2015	27.34	25.33	27.25	27.36	27.30	26.08	21.86	0
04/05/2015	27.54	25.57	27.51	27.56	27.49	26.10	21.86	0
05/05/2015	27.54	25.40	27.39	27.62	27.48	26.10	21.86	0
06/05/2015	27.45	25.43	27.45	27.62	27.46	26.09	21.86	0
07/05/2015	27.65	25.47	27.58	27.71	27.57	26.10	21.86	0
08/05/2015	28.01	25.76	27.96	27.97	27.90	26.10	21.86	0
09/05/2015	28.02	25.75	27.99	27.97	27.87	26.10	21.86	0
10/05/2015	27.92	25.64	27.92	27.99	27.76	26.10	21.86	0
11/05/2015	27.94	25.73	27.92	27.96	27.80	26.11	21.86	0
12/05/2015	27.79	25.68	27.84	27.82	27.74	26.11	21.87	0
13/05/2015	27.93	25.59	27.81	27.78	27.68	26.13	21.86	0
14/05/2015	27.88	25.68	27.90	27.81	27.76	26.15	21.86	0
15/05/2015	27.99	25.67	27.92	28.06	27.72	26.16	21.87	0
16/05/2015	28.05	25.63	27.97	27.95	27.78	26.14	21.86	0
17/05/2015	27.98	25.72	27.98	28.06	27.90	26.13	21.87	0.76
18/05/2015	28.08	25.73	28.02	28.17	28.01	26.14	21.86	0
19/05/2015	28.10	25.73	28.01	28.16	28.08	26.13	21.86	0
20/05/2015	28.18	25.72	28.07	28.24	28.13	26.11	21.86	0
21/05/2015	28.28	25.72	28.13	28.27	28.21	26.11	21.86	0
22/05/2015	28.36	25.76	28.23	28.28	28.24	26.10	21.86	0
23/05/2015	28.37	25.73	28.15	28.29	28.27	26.10	21.86	0
24/05/2015	28.49	25.74	28.26	28.28	28.34	26.10	21.86	0
25/05/2015	28.60	25.84	28.42	28.52	28.46	26.10	21.86	0
26/05/2015	28.62	25.84	28.45	28.50	28.44	26.10	21.86	0
27/05/2015	28.64	25.87	28.50	28.57	28.50	26.10	21.86	0.51
28/05/2015	28.53	25.81	28.42	28.43	28.46	26.10	21.86	0
29/05/2015	28.53	25.85	28.44	28.51	28.39	26.10	21.86	0
30/05/2015	28.53	25.79	28.42	28.40	28.47	26.10	21.86	0
31/05/2015	28.48	25.84	28.45	28.56	28.50	26.10	21.86	1.53

01/06/2015	28.35	25.81	28.38	28.57	28.44	26.10	21.86	9.14
02/06/2015	28.28	25.77	28.30	28.46	28.38	26.10	21.86	1.78
03/06/2015	28.22	25.76	28.23	28.38	28.31	26.10	21.86	0.25
04/06/2015	28.19	25.75	28.19	28.32	28.26	26.10	21.86	0.51
05/06/2015	28.19	25.71	28.13	28.28	28.27	26.10	21.86	0
06/06/2015	28.37	25.69	28.11	28.29	28.30	26.10	21.86	0.25
07/06/2015	28.70	25.79	28.32	28.37	28.42	26.09	21.86	0
08/06/2015	28.64	25.79	28.36	28.37	28.46	26.08	21.86	0
09/06/2015	28.64	25.75	28.31	28.31	28.45	26.09	21.86	0.25
10/06/2015	28.61	25.85	28.46	28.42	28.41	26.09	21.86	1.53
11/06/2015	28.47	25.83	28.41	28.36	28.33	26.09	21.86	27.94
12/06/2015	28.30	25.78	28.26	28.27	28.27	26.08	21.87	18.28
13/06/2015	26.99	25.50	26.60	27.60	27.26	26.02	22.01	132.85
14/06/2015	25.71	24.92	25.42	26.51	25.46	25.86	22.07	48.01
15/06/2015	25.78	24.84	25.65	26.37	25.40	25.81	21.96	0
16/06/2015	26.75	24.95	26.20	26.53	27.13	25.87	21.95	0
17/06/2015	27.58	25.11	26.71	26.73	27.60	25.93	21.94	0
18/06/2015	27.91	25.32	27.38	26.91	27.73	26.00	21.94	0
19/06/2015	27.94	25.37	27.47	27.01	27.82	26.00	21.94	0
20/06/2015	28.23	25.49	27.95	27.60	27.87	26.00	21.93	0
21/06/2015	28.16	25.56	27.96	27.36	27.98	26.01	21.94	12.7
22/06/2015	28.27	25.57	28.10	27.90	27.99	26.09	21.92	0.51
23/06/2015	28.29	25.57	28.02	27.70	28.15	26.06	21.89	1.01
24/06/2015	28.13	25.61	27.93	27.66	28.11	26.06	21.91	0
25/06/2015	28.25	25.51	28.03	27.76	28.12	26.07	21.90	0
26/06/2015	28.40	25.71	28.40	28.14	28.20	26.10	21.91	0
27/06/2015	28.30	25.69	28.30	28.25	28.20	26.10	21.91	0
28/06/2015	28.28	25.68	28.30	28.24	28.18	26.10	21.90	5.84
29/06/2015	28.25	25.72	28.32	28.33	28.12	26.10	21.90	0
30/06/2015	28.26	25.72	28.28	28.28	28.12	26.10	21.91	0
01/07/2015	28.23	25.77	28.41	28.45	28.14	26.10	21.90	5.85
02/07/2015	28.23	25.77	28.26	28.31	28.10	26.10	21.90	0
03/07/2015	28.33	25.77	28.37	28.39	28.08	26.10	21.90	0
04/07/2015	28.33	25.78	28.37	28.45	28.09	26.10	21.89	0
05/07/2015	28.43	25.80	28.44	28.46	28.13	26.10	21.88	5.33
06/07/2015	28.44	25.83	28.37	28.36	28.16	26.11	21.87	0
07/07/2015	28.34	25.71	28.28	28.34	28.10	26.10	21.87	0
08/07/2015	28.56	25.89	28.46	28.48	28.25	26.16	21.88	0
09/07/2015	28.53	25.88	28.51	28.58	28.30	26.13	21.87	2.54
10/07/2015	28.51	25.89	28.56	28.59	28.18	26.14	21.87	5.84
11/07/2015	28.29	25.77	28.29	28.46	28.05	26.11	21.87	0
12/07/2015	28.31	25.68	28.25	28.31	28.07	26.15	21.86	0
13/07/2015	28.72	25.95	28.63	28.74	28.37	26.17	21.86	0.51
14/07/2015	28.36	25.78	28.32	28.68	28.15	26.14	21.86	0
15/07/2015	28.48	25.87	28.38	28.55	28.21	26.19	21.86	0.25
16/07/2015	28.48	25.85	28.50	28.63	28.24	26.18	21.86	0.26
17/07/2015	28.46	25.85	28.42	28.43	28.26	26.19	21.86	4.82
18/07/2015	28.69	25.87	28.48	28.66	28.33	26.19	21.86	0
19/07/2015	28.73	25.89	28.56	28.60	28.40	26.20	21.86	0.51
20/07/2015	28.72	25.89	28.64	28.76	28.46	26.19	21.86	0

21/07/2015	28.77	25.95	28.72	28.72	28.45	26.20	21.86	0
22/07/2015	28.60	25.93	28.63	28.55	28.40	26.20	21.86	2.03
23/07/2015	28.89	25.96	28.75	28.79	28.49	26.20	21.86	0.26
24/07/2015	28.82	25.92	28.77	28.73	28.47	26.19	21.86	0
25/07/2015	28.95	25.98	28.84	28.91	28.47	26.20	21.86	0
26/07/2015	28.93	25.88	28.60	28.64	28.44	26.19	21.86	0
27/07/2015	29.01	26.01	28.85	28.91	28.79	26.20	21.86	0
28/07/2015	29.01	26.02	28.91	29.01	28.88	26.20	21.86	0
29/07/2015	29.06	26.06	28.92	29.03	28.80	26.20	21.86	0
30/07/2015	28.85	26.00	28.83	28.85	28.62	26.19	21.86	0
31/07/2015	28.84	25.98	28.76	28.85	28.73	26.20	21.85	0
01/08/2015	28.96	25.99	28.78	28.84	28.94	26.19	21.86	0
02/08/2015	28.99	26.02	28.88	28.98	28.84	26.19	21.86	0
03/08/2015	28.99	26.08	28.84	28.99	28.83	26.19	21.86	0
04/08/2015	29.23	26.11	29.07	29.17	28.91	26.18	21.86	0
05/08/2015	29.10	26.21	29.14	29.09	28.82	26.19	21.86	5.08
06/08/2015	29.09	26.22	29.08	29.06	28.82	26.20	21.85	0
07/08/2015	28.80	26.17	28.88	28.81	28.68	26.19	21.85	0.76
08/08/2015	28.80	26.15	28.84	28.91	28.66	26.20	21.86	0
09/08/2015	28.94	26.13	28.90	28.95	28.71	26.20	21.86	0
10/08/2015	29.06	26.16	28.94	29.02	28.89	26.20	21.85	6.35
11/08/2015	29.04	26.07	28.94	29.14	28.82	26.20	21.86	0.51
12/08/2015	28.97	26.09	28.89	28.96	28.92	26.20	21.85	0
13/08/2015	29.17	26.17	29.04	29.12	28.92	26.20	21.85	0
14/08/2015	29.13	26.21	29.16	29.19	28.85	26.20	21.85	0
15/08/2015	29.09	26.27	29.08	29.16	28.95	26.20	21.85	0.25
16/08/2015	29.19	26.28	29.11	29.10	28.89	26.20	21.86	0
17/08/2015	29.36	26.42	29.36	29.34	29.04	26.20	21.86	0
18/08/2015	29.49	26.44	29.42	29.36	29.44	26.20	21.85	0
19/08/2015	29.54	26.47	29.57	29.55	29.56	26.20	21.86	0
20/08/2015	29.46	26.38	29.44	29.36	29.45	26.20	21.86	0
21/08/2015	29.25	26.29	29.26	29.21	29.17	26.20	21.86	0
22/08/2015	29.26	26.20	29.18	29.18	29.28	26.20	21.86	0
23/08/2015	29.25	26.21	29.20	29.21	29.42	26.20	21.86	0.76
24/08/2015	29.39	26.21	29.23	29.35	29.43	26.20	21.86	3.56
25/08/2015	29.36	26.20	29.23	29.28	29.41	26.20	21.86	17.02
26/08/2015	29.60	26.17	29.21	29.15	29.46	26.20	21.86	0.25
27/08/2015	29.62	26.16	29.20	29.14	29.56	26.20	21.87	32.26
28/08/2015	29.65	26.18	29.30	29.16	29.59	26.20	21.86	20.57
29/08/2015	29.55	26.23	29.30	29.26	29.52	26.20	21.86	0
30/08/2015	29.56	26.22	29.34	29.40	29.48	26.20	21.87	45.22
31/08/2015	29.51	26.21	29.26	29.40	29.41	26.20	21.89	44.95
01/09/2015	29.39	26.18	29.20	29.37	29.38	26.20	21.86	0.51
02/09/2015	29.56	26.19	29.28	29.52	29.34	26.20	21.86	1.78
03/09/2015	29.88	26.33	29.52	29.68	29.58	26.19	21.86	0
04/09/2015	29.94	26.37	29.76	29.77	29.74	26.20	21.86	0
05/09/2015	29.94	26.41	29.77	29.82	29.71	26.20	21.86	0
06/09/2015	29.91	26.42	29.81	29.84	29.68	26.20	21.86	0
07/09/2015	29.78	26.54	29.73	29.79	29.67	26.20	21.86	17.53
08/09/2015	29.59	26.47	29.61	29.57	29.59	26.20	21.86	0

09/09/2015	29.60	26.48	29.58	29.61	29.50		26.20	21.86	0.25
10/09/2015	29.59	26.49	29.59	29.62	29.39		26.20	21.86	0
11/09/2015	29.67	26.46	29.57	29.62	29.45		26.20	21.86	0
12/09/2015	29.82	26.52	29.69	29.80	29.65		26.20	21.86	0
13/09/2015	29.67	26.38	29.60	29.72	29.54		26.20	21.86	0
14/09/2015	29.68	26.44	29.61	29.75	29.50		26.20	21.86	0
15/09/2015	29.76	26.36	29.61	29.72	29.55		26.20	21.86	0.51
16/09/2015	29.76	26.40	29.67	29.75	29.59		26.20	21.86	0
17/09/2015	29.73	26.37	29.63	29.67	29.64		26.20	21.86	0
18/09/2015	29.82	26.49	29.80	29.79	29.78		26.20	21.86	0
19/09/2015	29.78	26.31	29.52	29.78	29.74		26.20	21.86	37.08
20/09/2015	29.77	26.28	29.46	29.65	29.64		26.20	21.86	0
21/09/2015	29.75	26.36	29.62	29.70	29.60		26.20	21.86	0
22/09/2015	29.81	26.43	29.65	29.73	29.61		26.20	21.86	12.96
23/09/2015	29.54	26.33	29.54	29.62	29.54		26.20	21.86	17.02
24/09/2015	29.45	26.35	29.37	29.45	29.40		26.20	21.86	9.39
25/09/2015	29.49	26.34	29.40	29.44	29.33		26.20	21.86	3.82
26/09/2015	29.32	26.20	29.17	29.40	29.14		26.20	21.89	41.39
27/09/2015	28.13	25.61	27.51	28.26	27.83		26.19	22.02	50.55
28/09/2015	28.43	25.54	27.83	28.10	28.79		26.13	21.95	0.25
29/09/2015	28.91	25.75	28.50	28.45	28.86		26.12	21.88	0.77
30/09/2015	29.24	26.00	28.98	28.78	29.03		26.14	21.86	0
01/10/2015	29.33	26.08	29.15	29.01	29.11		26.14	21.86	0
02/10/2015	29.47	26.11	29.26	28.98	29.20		26.16	21.86	26.41
03/10/2015	29.21	26.03	28.71	28.70	29.18		26.16	21.86	0
04/10/2015	29.14	25.98	29.03	28.93	29.05		26.18	21.86	6.35
05/10/2015	29.21	26.18	29.11	29.06	29.06		26.20	21.86	0
06/10/2015	29.61	26.28	29.48	29.52	29.43		26.20	21.87	0.51
07/10/2015	29.72	26.45	29.65	29.65	29.53		26.20	21.86	0
08/10/2015	29.77	26.44	29.76	29.88	29.64		26.20	21.86	0.51
09/10/2015	29.73	26.47	29.65	29.69	29.54		26.20	21.86	0
10/10/2015	29.77	26.43	29.68	29.77	29.59		26.20	21.86	0.25
11/10/2015	29.90	26.46	29.80	29.95	29.69		26.20	21.89	55.63
12/10/2015	29.89	26.43	29.78	29.90	29.75		26.20	21.87	0
13/10/2015	29.88	26.36	29.69	29.85	29.80		26.20	21.90	5.33
14/10/2015	29.93	26.26	29.63	29.75	29.81		26.20	21.90	6.61
15/10/2015	29.72	26.15	29.30	29.52	29.50		26.20	21.90	12.96
16/10/2015	29.29	25.96	28.79	29.13	29.34		26.20	21.91	29.96
17/10/2015	28.43	25.64	27.89	28.27	29.12		26.13	22.02	75.7
18/10/2015	26.78	25.31	26.31	27.29	28.37		26.08	22.05	34.04
19/10/2015					26.50		25.92	22.11	55.12
20/10/2015	25.53	24.99	25.15		25.36		25.77	22.08	15.74
21/10/2015	26.16	25.00	25.77	26.52	25.27		25.71	22.05	0
22/10/2015	27.11	25.07	26.14	26.66	27.55		25.73	22.05	17.02
23/10/2015	27.78	25.17	26.52	26.81	28.12		25.78	22.05	2.28
24/10/2015	27.44	25.12	26.33	26.78			25.80	22.04	0.51
25/10/2015	27.99	25.26	26.93	26.94	28.55		25.80	22.04	0
26/10/2015	28.22	25.33	27.34	27.13	28.48	24.86	25.82	22.03	0.76
27/10/2015	28.38	25.36	27.31	27.24	28.53	24.84	25.84	22.03	2.03
28/10/2015	28.67	25.51	27.90	27.48	28.66	24.84	25.88	22.03	0.51

29/10/2015	28.87	25.67	28.38	27.59	28.72	24.84	25.90	22.01	0
30/10/2015	29.12	25.80	28.89	27.84	28.90	24.84	25.90	21.99	1.02
31/10/2015	29.25	25.95	29.18	28.66	29.06	24.82	25.90	21.98	0
01/11/2015	29.22	25.97	29.20	28.92	29.08	24.83	25.90	21.95	0
02/11/2015	29.35	26.06	29.29	29.28	29.11	24.83	25.92	21.95	0
03/11/2015	29.30	26.05	29.29	29.24	29.14	24.83	25.96	21.95	0.25
04/11/2015	29.35	26.10	29.32	29.33	29.14	24.84	25.98	21.95	27.94
05/11/2015	28.85	26.03	28.61	29.04	28.96	24.85	25.97	21.97	59.19
06/11/2015	27.98	25.43	27.42	27.78	28.46	24.93	25.90	21.98	2.28
07/11/2015	28.16	25.43	27.71	27.54	28.98	24.84	25.86	21.97	14.23
08/11/2015	28.72	25.64	28.45	27.88	28.85	24.82	25.89	21.95	0
09/11/2015	28.88	25.75	28.75	27.97	28.82	24.79	25.90	21.95	0
10/11/2015	29.02	25.87	28.95	28.42	28.88	24.77	25.91	21.95	0
11/11/2015	28.93	25.80	28.82	28.29	28.91	24.77	25.92	21.95	7.11
12/11/2015	28.98	25.86	28.92	28.43	28.94	24.79	25.92	21.95	15.24
13/11/2015	28.91	25.83	28.85	28.34	28.94	24.82	25.92	21.95	39.36
14/11/2015	28.72	25.78	28.56	28.00	28.96	24.82	25.92	21.95	12.21
15/11/2015	26.40	25.14	25.83	26.92	27.43	24.94	25.83	22.03	28.44
16/11/2015	26.39	25.03	26.22	26.78	28.34	24.83	25.81	21.95	2.03
17/11/2015	26.62	25.02	26.27	26.65	28.58	24.76	25.81	21.97	2.29
18/11/2015	27.19	25.10	26.50	26.70	28.47	24.76	25.81	21.95	1.52
19/11/2015	27.80	25.18	26.98	26.84	28.43	24.77	25.81	21.95	0.51
20/11/2015	28.11	25.30	27.35	27.04	28.48	24.75	25.85	21.95	0
21/11/2015	28.37	25.50	28.02	27.27	28.61	24.74	25.89	21.95	10.92
22/11/2015	28.50	25.58	28.28	27.66	28.59	24.75	25.89	21.95	15.75
23/11/2015	28.41	25.61	28.33	27.53	28.56	24.75	25.90	21.95	13.21
24/11/2015	27.59	25.23	26.60	26.99	28.38	24.80	25.90	22.01	31.49
25/11/2015	26.97	25.06	26.28	26.71	28.38	24.85	25.81	21.99	5.59
26/11/2015	27.30	25.07	26.41	26.72	28.51	24.78	25.81	21.95	0
27/11/2015	27.37	25.12	26.69	26.79	28.45	24.78	25.82	21.95	0
28/11/2015	27.29	25.14	26.69	26.82	28.45	24.76	25.84	21.95	0
29/11/2015	27.81	25.28	27.41	27.04	28.42	24.75	25.88	21.95	0.76
30/11/2015	28.20	25.55	28.19	27.21	28.43	24.74	25.90	21.95	1.77
01/12/2015	28.23	25.56	28.25	27.35	28.45	24.74	25.90	21.95	4.84
02/12/2015	28.31	25.59	28.29	27.71	28.43	24.74	25.90	21.95	0.25
03/12/2015	28.06	25.61	28.22	27.51	28.47	24.74	25.90	21.95	27.69
04/12/2015	27.50	25.36	27.38	27.15	28.39	24.75	25.90	21.99	9.64
05/12/2015	26.32	25.07	26.12	26.71	28.24	24.75	25.85	22.05	32.26
06/12/2015	26.22	24.93	25.99	26.46	28.17	24.79	25.81	21.97	0.25
07/12/2015	27.00	25.02	26.42	26.54	28.28	24.74	25.81	21.95	3.3
08/12/2015	26.31	24.95	25.76	26.42	28.21	24.74	25.81	21.98	16.76
09/12/2015	26.24	24.91	25.85	26.22	28.19	24.74	25.81	21.98	0.26
10/12/2015	26.73	24.95	26.28	26.32	28.18	24.74	25.81	21.97	0
11/12/2015	27.46	25.20	27.10	26.59	28.18	24.74	25.82	21.95	0
12/12/2015	27.61	25.32	27.50	26.71	28.18	24.74	25.84	21.96	0
13/12/2015	27.88	25.44	27.88	26.95	28.20	24.74	25.86	21.95	0
14/12/2015	27.82	25.46	27.88	26.90	28.25	24.74	25.88	21.95	0
15/12/2015	27.90	25.48	27.99	27.00	28.26	24.74	25.89	21.95	0
16/12/2015	28.05	25.55	28.22	27.32	28.28	24.74	25.90	21.95	0.25
17/12/2015	28.17	25.62	28.29	28.02	28.30	24.74	25.90	21.95	1.02

18/12/2015	27.98	25.60	28.20	27.76	28.32	24.74	25.90	21.95	1.01
19/12/2015	27.73	25.48	27.92	27.47	28.26	24.74	25.90	21.95	6.86
20/12/2015	27.90	25.52	28.01	27.69	28.20	24.74	25.90	21.95	1.52
21/12/2015	27.90	25.56	28.09	27.75	28.24	24.74	25.90	21.95	1.02
22/12/2015	27.92	25.56	28.12	27.79	28.15	24.74	25.90	21.95	0.26
23/12/2015	27.92	25.60	28.14	27.95	28.13	24.74	25.90	21.95	4.83
24/12/2015	27.98	25.63	28.17	28.11	28.14	24.74	25.90	21.96	6.09
25/12/2015	27.85	25.58	28.10	27.99	28.12	24.74	25.90	21.96	0
26/12/2015	28.03	25.64	28.14	28.18	28.13	24.74	25.91	21.95	1.27
27/12/2015	27.88	25.59	28.08	28.10	28.16	24.74	25.91	21.95	0
28/12/2015	27.98	25.61	28.07	28.09	28.18	24.74	25.91	21.95	2.03
29/12/2015	28.01	25.61	28.05	28.07	28.18	24.74	25.92	21.95	1.78
30/12/2015	27.86	25.57	28.03	28.06	28.19	24.74	25.91	21.95	1.78
31/12/2015	27.87	25.56	28.02	28.05	28.13	24.74	25.91	21.95	0
01/01/2016	27.97	25.56	28.06	28.00	28.10	24.74	25.91	21.95	0
02/01/2016	28.06	25.61	28.07	28.00	28.05	24.74	25.92	21.95	3.56
03/01/2016	28.09	25.64	28.10	28.06	28.03	24.75	25.92	21.95	0
04/01/2016	27.96	25.62	28.05	28.16	28.03	24.76	25.96	21.95	1.26
05/01/2016	27.51	25.51	27.64	27.62	27.78	24.80	25.98	21.95	1.78
06/01/2016	27.62	25.47	27.64	27.62	27.82	24.82	25.99	21.95	0
07/01/2016	27.75	25.55	27.78	27.80	27.84	24.84	26.00	21.95	0
08/01/2016	27.83	25.58	27.82	27.85	27.86	24.84	26.00	21.96	0
09/01/2016	27.91	25.61	27.91	27.96	27.85	24.84	26.00	21.95	0
10/01/2016	27.89	25.63	27.95	27.99	27.89	24.84	26.00	21.95	1.78
11/01/2016	27.40	25.49	27.62	27.80	27.81	24.86	26.00	21.95	20.07
12/01/2016	27.59	25.50	27.62	27.63	27.79	24.84	26.00	21.95	34.29
13/01/2016	27.04	25.43	26.89	27.54	27.52	24.95	25.97	22.08	133.09
14/01/2016	25.07	24.73	24.65	26.25	25.29	24.94	25.74	22.23	10.16
15/01/2016	25.57	24.76	25.38	26.36	24.91	24.76	25.71	22.08	0
16/01/2016	26.29	24.84	26.06	26.47	26.70	24.71	25.71	22.05	0
17/01/2016	26.92	25.02	26.55	26.60	27.65	24.65	25.71	22.05	4.07
18/01/2016	26.74	25.01	26.67	26.79	27.58	24.66	25.71	22.03	0
19/01/2016	26.78	25.08	26.76	26.96	27.57	24.64	25.75	22.01	3.3
20/01/2016	27.03	25.15	27.05	27.02	27.57	24.65	25.77	21.98	0
21/01/2016	27.09	25.25	27.25	27.14	27.55	24.66	25.80	21.97	1.78
22/01/2016	27.05	25.28	27.29	27.27	27.58	24.66	25.82	21.95	0
23/01/2016	26.55	25.13	26.91	27.17	27.50	24.71	25.82	21.96	0
24/01/2016	26.71	25.17	26.84	27.05	27.29	24.71	25.85	21.95	0
25/01/2016	27.06	25.26	27.19	27.22	27.37	24.70	25.89	21.95	0.25
26/01/2016	27.22	25.34	27.24	27.28	27.36	24.74	25.90	21.95	0
27/01/2016	27.30	25.33	27.33	27.36	27.46	24.74	25.90	21.95	0
28/01/2016	27.21	25.34	27.33	27.43	27.53	24.74	25.90	21.95	11.43
29/01/2016	26.79	25.22	27.10	27.43	27.62	24.74	25.89	21.95	0
30/01/2016	26.93	25.23	26.96	27.06	27.24	24.74	25.90	21.95	0
31/01/2016	27.13	25.24	27.20	27.29	27.46	24.74	25.90	21.95	0
01/02/2016	27.13	25.31	27.21	27.29	27.41	24.74	25.90	21.95	0
02/02/2016	27.24	25.28	27.20	27.21	27.38	24.74	25.90	21.95	0
03/02/2016	27.36	25.36	27.33	27.30	27.39	24.74	25.90	21.95	0
04/02/2016	27.31	25.32	27.35	27.38	27.49	24.74	25.90	21.95	2.8
05/02/2016	26.65	25.20	26.92	27.40	27.38	24.74	25.88	21.95	10.66

06/02/2016	26.77	25.21	26.86	27.00	27.27	24.74	25.90	21.95	0
07/02/2016	26.49	25.17	26.87	26.96	27.18	24.74	25.91	21.95	0
08/02/2016	26.50	25.18	26.73	26.78	27.11	24.74	25.92	21.94	0
09/02/2016	26.34	25.14	26.66	26.70	27.14	24.75	25.93	21.93	0
10/02/2016	26.32	25.12	26.57	26.71	26.90	24.74	25.94	21.95	0.26
11/02/2016	26.23	25.09	26.51	26.68	27.07	24.74	25.95	21.93	0
12/02/2016	26.59	25.13	26.65	26.68	27.07	24.74	25.99	21.94	0
13/02/2016	26.70	25.17	26.73	26.77	27.07	24.74	25.98	21.93	0
14/02/2016	26.75	25.17	26.74	26.74	27.04	24.78	25.98	21.93	0.76
15/02/2016	26.82	25.17	26.78	26.75	27.06	24.82	25.97	21.92	0.77
16/02/2016	26.79	25.18	26.82	26.84	27.19	24.84	25.93	21.93	12.95
17/02/2016	26.77	25.18	26.83	26.82	27.13	24.83	25.91	21.93	42.92
18/02/2016	26.70	25.19	26.85	26.84	27.10	24.84	25.92	21.91	0
19/02/2016	26.70	25.19	26.84	26.82	27.12	24.83	25.90	21.93	0.25
20/02/2016	26.65	25.19	26.82	26.82	27.08	24.82	25.92	21.91	3.05
21/02/2016	26.68	25.20	26.85	26.83	27.12	24.81	25.91	21.91	0
22/02/2016	26.72	25.22	26.84	26.82	27.11	24.80	25.92	21.91	0.77
23/02/2016	26.81	25.22	26.87	26.86	27.12	24.80	25.92	21.90	0.5
24/02/2016	26.77	25.23	26.88	26.88	27.10	24.78	25.91	21.93	1.78
25/02/2016	26.52	25.14	26.81	26.94	27.25	24.81	25.90	21.93	0
26/02/2016	26.35	25.14	26.67	26.85	27.25	24.78	25.90	21.92	1.02
27/02/2016	26.21	25.13	26.61	26.42	27.17	24.81	25.90	21.89	1.78
28/02/2016	26.44	25.12	26.53	26.43	27.14	24.83	25.90	21.89	0
29/02/2016	26.54	25.13	26.64	26.48	27.06	24.83	25.90	21.89	0
01/03/2016	26.64	25.13	26.64	26.57	27.03	24.83	25.90	21.88	0
02/03/2016	26.67	25.13	26.68	26.65	27.13	24.84	25.90	21.91	0
03/03/2016	26.63	25.13	26.71	26.66	27.10	24.83	25.90	21.89	6.08
04/03/2016	26.63	25.14	26.75	26.75	27.12	24.83	25.90	21.90	1.53
05/03/2016	26.73	25.16	26.78	26.76	27.07	24.82	25.90	21.89	7.11
06/03/2016	26.67	25.17	26.83	26.79	27.05	24.81	25.90	21.91	0
07/03/2016	26.58	25.17	26.79	26.77	26.99	24.79	25.90	21.90	7.37
08/03/2016	26.54	25.17	26.77	26.84	26.92	24.76	25.89	21.91	9.4
09/03/2016	26.58	25.18	26.78	26.75	26.80	24.75	25.90	21.91	0
10/03/2016	26.58	25.17	26.79	26.81	26.88	24.75	25.87	21.91	0
11/03/2016	26.58	25.17	26.82	26.91	26.96	24.76	25.86	21.90	0
12/03/2016	26.59	25.18	26.85	26.90	27.01	24.74	25.85	21.90	0
13/03/2016	26.71	25.18	26.86	26.87	27.07	24.75	25.84	21.88	0
14/03/2016	26.74	25.20	26.92	26.90	27.08	24.74	25.85	21.89	0
15/03/2016	26.87	25.19	26.91	26.92	27.10	24.74	25.84	21.87	0
16/03/2016	26.95	25.22	26.97	26.95	27.10	24.74	25.84	21.87	0
17/03/2016	26.99	25.24	27.01	26.98	27.07	24.74	25.86	21.89	0
18/03/2016	26.99	25.26	27.03	26.99	27.09	24.74	25.87	21.90	0
19/03/2016	27.09	25.29	27.09	27.07	27.10	24.74	25.88	21.89	3.31
20/03/2016	27.11	25.28	27.14	27.20	27.14	24.74	25.84	21.90	32.76
21/03/2016	26.82	25.24	27.02	27.32	27.18	24.74	25.83	21.91	0.5
22/03/2016	26.44	25.16	26.78	27.22	26.98	24.74	25.82	21.89	0
23/03/2016	26.71	25.17	26.87	27.24	27.06	24.74	25.81	21.89	0
24/03/2016	26.83	25.21	26.98	27.25	27.07	24.74	25.81	21.88	0
25/03/2016	26.92	25.21	27.00	27.30	27.15	24.74	25.81	21.88	0
26/03/2016	27.01	25.27	27.12	27.17	27.13	24.74	25.81	21.87	0

27/03/2016	27.13	25.30	27.17	27.18	27.20	24.74	25.81	21.86	0
28/03/2016	26.65	25.23	26.96	27.37	27.17	24.81	25.81	21.90	0
29/03/2016	26.58	25.13	26.74	27.28	27.17	24.78	25.81	21.88	0
30/03/2016	26.83	25.09	26.74	27.24	27.25	24.74	25.81	21.86	0
31/03/2016	27.25	25.30	27.20	27.26	27.26	24.74	25.81	21.87	0
01/04/2016	26.77	25.16	26.81	27.24	27.23	24.79	25.81	21.89	0
02/04/2016	26.42	25.03	26.52	27.10	27.26	24.74	25.80	21.89	0.51
03/04/2016	26.51	25.03	26.59	27.04	27.13	24.74	25.78	21.88	4.32
04/04/2016	27.13	25.27	27.18	27.24	27.23	24.74	25.81	21.90	12.44
05/04/2016	26.94	25.21	26.97	27.17	27.13	24.74	25.80	21.89	0
06/04/2016	26.68	25.12	26.73	27.16	27.07	24.77	25.80	21.89	0
07/04/2016	26.93	25.19	26.98	27.23	27.06	24.78	25.81	21.89	0
08/04/2016	27.23	25.36	27.28	27.30	27.18	24.74	25.83	21.88	0
09/04/2016	27.14	25.36	27.24	27.40	27.10	24.76	25.84	21.90	0
10/04/2016	27.22	25.34	27.24	27.33	27.10	24.75	25.85	21.88	0
11/04/2016	27.28	25.35	27.31	27.39	27.25	24.74	25.86	21.87	0
12/04/2016	27.11	25.33	27.25	27.40	27.31	24.74	25.81	21.87	0
13/04/2016	27.60	25.42	27.46	27.50	27.33	24.74	25.86	21.86	0
14/04/2016	27.71	25.49	27.69	27.73	27.47	24.74	25.84	21.86	0
15/04/2016	27.58	25.43	27.60	27.61	27.47	24.74	25.85	21.87	0
16/04/2016	27.64	25.49	27.66	27.88	27.49	24.75	25.84	21.88	0
17/04/2016	27.42	25.37	27.48	27.56	27.46	24.75	25.86	21.87	0
18/04/2016	27.53	25.48	27.57	27.79	27.52	24.74	25.87	21.88	0
19/04/2016	26.91	25.23	26.96	27.43	27.40	24.77	25.81	21.88	0
20/04/2016	26.78	25.12	26.76	27.32	27.44	24.74	25.81	21.86	0
21/04/2016	27.37	25.31	27.29	27.41	27.35	24.74	25.86	21.86	0
22/04/2016	27.64	25.52	27.61	27.74	27.53	24.74	25.90	21.87	0
23/04/2016	27.38	25.36	27.36	27.61	27.41	24.74	25.87	21.87	0
24/04/2016	27.04	25.22	26.96	27.46	27.51	24.74	25.82	21.87	0
25/04/2016	27.47	25.32	27.33	27.57	27.39	24.74	25.88	21.86	0
26/04/2016	27.89	25.61	27.82	27.86	27.70	24.74	25.90	21.86	0
27/04/2016	28.03	25.66	27.96	27.93	27.78	24.75	25.90	21.86	0
28/04/2016	28.02	25.54	27.83	28.04	27.74	24.82	25.90	21.86	0
29/04/2016	27.95	25.44	27.59	27.78	27.73	24.77	25.90	21.86	0
30/04/2016	28.08	25.70	27.97	28.01	27.92	24.74	25.90	21.87	0
01/05/2016	28.23	25.79	28.21	28.21	28.13	24.75	25.91	21.87	0
02/05/2016	28.15	25.75	28.12	28.09	28.06	24.79	25.90	21.88	0
03/05/2016	28.28	25.70	28.14	28.16	28.02	24.83	25.90	21.87	0
04/05/2016	28.19	25.64	28.02	28.05	27.96	24.82	25.90	21.88	28.96
05/05/2016	28.00	25.55	27.95	27.98	27.98	24.82	25.89	21.88	0
06/05/2016	27.90	25.71	28.04	27.95	27.93	24.82	25.90	21.88	0
07/05/2016	28.06	25.70	28.07	28.11	27.98	24.82	25.90	21.88	0
08/05/2016	28.12	25.72	28.08	28.19	28.07	24.79	25.90	21.87	0.25
09/05/2016	28.14	25.74	28.10	28.23	28.06	24.79	25.90	21.87	0
10/05/2016	28.11	25.69	28.03	28.10	28.00	24.83	25.90	21.86	0
11/05/2016	28.29	25.73	28.23	28.23	28.13	24.83	25.90	21.86	4.57
12/05/2016	28.45	25.78	28.32	28.37	28.29	24.83	25.90	21.86	0
13/05/2016	28.58	25.84	28.48	28.47	28.30	24.84	25.90	21.86	0
14/05/2016	28.62	25.87	28.57	28.55	28.38	24.84	25.90	21.86	4.83
15/05/2016	28.58	25.88	28.59	28.50	28.44	24.84	25.90	21.86	0

16/05/2016	28.62	25.93	28.60	28.56	28.41	24.84	25.90	21.86	0
17/05/2016	28.61	25.91	28.59	28.61	28.43	24.84	25.90	21.86	0
18/05/2016	28.64	25.92	28.63	28.60	28.44	24.84	25.90	21.86	0
19/05/2016	28.75	25.95	28.75	28.73	28.50	24.84	25.90	21.86	0
20/05/2016	28.74	25.96	28.73	28.73	28.51	24.84	25.90	21.86	0
21/05/2016	28.75	25.92	28.75	28.75	28.50	24.87	25.90	21.86	0
22/05/2016	28.85	25.97	28.80	28.78	28.58	24.85	25.90	21.87	0
23/05/2016	28.86	26.04	28.85	28.87	28.66	24.85	25.90	21.86	0
24/05/2016	28.92	26.04	28.88	28.88	28.75	24.86	25.90	21.86	0
25/05/2016	29.04	26.04	28.98	28.98	28.82	24.90	25.90	21.86	0
26/05/2016	29.03	26.01	28.94	28.99	28.82	24.93	25.90	21.86	0.51
27/05/2016	29.10	26.04	29.03	28.97	28.88	24.93	25.90	21.86	0
28/05/2016	29.18	26.10	29.14	29.09	28.90	24.92	25.90	21.86	0
29/05/2016	29.23	26.08	29.13	29.08	28.91	24.93	25.90	21.86	0
30/05/2016	29.11	26.09	29.16	29.09	28.87	24.93	25.90	21.87	0
31/05/2016	29.13	26.10	29.14	29.08	28.94	24.93	25.90	21.87	0
01/06/2016	29.20	26.18	29.19	29.17	29.00	24.93	25.90	21.88	0
02/06/2016	29.14	26.12	29.14	29.15	28.88	24.93	25.90	21.88	0
03/06/2016	29.14	26.12	29.14	29.17	28.85	24.93	25.91	21.87	0.76
04/06/2016	29.05	26.14	29.09	29.17	28.84	24.93	25.92	21.86	20.06
05/06/2016	29.05	26.12	29.01	29.13	28.87	24.93	25.91	21.86	1.27
06/06/2016	28.83	25.95	28.81	28.92	28.80	24.93	25.90	21.86	3.81
07/06/2016	28.98	25.93	28.81	28.87	28.86	24.93	25.90	21.86	0
08/06/2016	29.00	25.98	28.85	28.92	28.87	24.92	25.90	21.86	4.32
09/06/2016	29.06	26.00	28.95	29.01	28.93	24.93	25.90	21.86	0
10/06/2016	29.20	26.03	28.99	29.07	29.02	24.93	25.90	21.86	0
11/06/2016	29.32	26.07	29.12	29.13	29.10	24.93	25.90	21.86	0
12/06/2016	29.43	26.10	29.24	29.22	29.17	24.93	25.90	21.86	0
13/06/2016	29.55	26.17	29.38	29.32	29.22	24.93	25.90	21.86	0
14/06/2016	29.50	26.23	29.43	29.34	29.29	24.93	25.90	21.86	0
15/06/2016	29.48	26.22	29.42	29.38	29.28	24.93	25.90	21.86	1.02
16/06/2016	29.33	26.18	29.31	29.31	29.19	24.93	25.90	21.86	22.36
17/06/2016	29.18	26.12	29.19	29.20	29.10	24.94	25.90	21.86	11.16
18/06/2016	28.88	26.04	28.95	28.99	28.97	24.95	25.90	21.86	14.23
19/06/2016	28.73	25.96	28.78	28.78	28.84	24.97	25.90	21.86	0
20/06/2016	28.83	25.94	28.77	28.84	28.78	24.98	25.90	21.86	0
21/06/2016	29.00	26.01	28.90	28.94	28.83	25.00	25.90	21.86	0
22/06/2016	29.10	26.06	29.04	29.06	28.90	24.97	25.90	21.86	2.55
23/06/2016	28.99	25.98	28.87	29.05	28.91	25.01	25.90	21.86	19.81
24/06/2016	28.97	25.96	28.88	28.95	28.87	24.99	25.90	21.86	0.25
25/06/2016	29.07	26.07	29.04	29.00	28.86	24.95	25.90	21.86	7.11
26/06/2016	29.19	26.14	29.14	29.11	28.96	25.00	25.90	21.86	0
27/06/2016	29.10	26.19	29.25	29.13	29.01	25.03	25.90	21.86	0
28/06/2016				29.09	28.97	25.03	25.90	21.86	0
29/06/2016				29.09	28.97	25.03	25.90	21.86	3.56
30/06/2016					29.03	25.03	25.90	21.86	0
01/07/2016					29.06	25.02		21.87	0
02/07/2016								21.86	0
03/07/2016								21.86	0
04/07/2016								21.86	0
