# Supplementary material for: Ecological patterns in anchialine caves
Source: PLoS One. 2018 Nov 7;13(11):e0202909. doi: 10.1371/journal.pone.0202909 (PMC6221257; doi:10.1371/journal.pone.0202909)
Supplement: S3 Table — Stygobitic species (+). (DOCX) [file pone.0202909.s006.docx]

**S3 Table. - Population estimation size (mean and [+/-] standard deviation) and presence (*) of macrofauna in the anchialine caves of Cozumel.** (¶, **&**) taxa which were identified as one during the ecological census. (B) Species recorded in the bibliography, but not observed in this study.

| **Taxa** | **Population estimation size / presence** | | | | | | |
| --- | --- | --- | --- | --- | --- | --- | --- |
|  | El Aerolito | La Quebrada | | | Tres Potrillos | Bambú | |
| **Porifera** |  | |  |  | | |  |
| *Acarnus innominatus* Gray, 1867 | 1294+/-2708 | |  |  | | |  |
| *Aciculites higginsii* Schmidt, 1879 | 1757+/-2785 ¶ | |  |  | | |  |
| Agelasidae | 11294+/-20587 | |  |  | | |  |
| *Alectona* sp*.* | * | |  |  | | |  |
| Calcarea sp. 1 | * | |  |  | | |  |
| Calcarea sp. 2 | 27009+/-26195 | |  |  | | |  |
| Calcarea sp. 3 | * | |  |  | | |  |
| Calcarea sp. 4 | 811+/-1405 | |  |  | | |  |
| Chondrosiida | 442+/-885 | |  |  | | |  |
| *Cinachyrella kuekenthali* (Uliczka, 1929) | | | * |  | | |  |
| *Dendroxea* sp*.* |  | | * |  | | |  |
| *Dercitus* sp*.* | * | |  |  | | |  |
| *Diplastrella megastellata* Hechtel, 1965 | 6862+/-11292 | |  |  | | |  |
| *Diplastrella* sp*.* |  | | 6486+/-1801 |  | | |  |
| *Discodermia adhaerens* Van Soest, Meesters & Becking, 2014 | 493+/-519 | | 1297+/-782 |  | | |  |
| *Gastrophanella* sp*.* | 1757+/-2785 ¶ | |  |  | | |  |
| *Geodia neptuni* (Sollas, 1886) | 18570+/-16007 | |  |  | | |  |
| *Geodia* sp. 1 | 5390+/-5751 | |  |  | | |  |
| *Geodia* sp. 2 | 3060+/-5374 | |  |  | | |  |
| *Haliclona (Reniera)* sp. 1 | * | |  |  | | |  |
| *Haliclona (Reniera)* sp. 2 | | | 9729+/-3079 |  | | |  |
| *Leiodermatium* sp*.* | * | |  |  | | |  |
| *Lithobactrum* sp*.* |  | | * |  | | |  |
| *Microscleroderma* sp*.* | * | |  |  | | |  |
| *Placospongia* sp*.* | 5146+/-5091 | |  |  | | |  |
| *Plakinastrella onkodes* Uliczka, 1929 | * | | * |  | | |  |
| *Plakortis angulospiculatus* (Carter, 1879) | 540+/-468 | |  |  | | |  |
| *Plakortis* sp*.* | 442+/-885 | |  |  | | |  |
| *Pleroma* sp*.* |  | | * |  | | |  |
| *Psammastra*sp*.* | * | |  |  | | |  |
| *Stelletta* sp*.* | 39+/-92 | |  |  | | |  |
| *Stelletta* sp. 2 | * | |  |  | | |  |
| *Tethya* sp. 1 | 2270+/-3699 & | |  |  | | |  |
| *Tethya* sp. 2 | 2270+/-3699 & | |  |  | | |  |
| *Tethya* sp. 3 | * | |  |  | | |  |
| **Cnidaria** |  | |  |  | | |  |
| Actiniaria | 270+/-468 | |  |  | | |  |
| *Balanophyllia (Balanophyllia) bayeri* Cairns, 1979 | 3984+/-7969 | |  |  | | |  |
| *Corynactis* cf*. caribbeorum* (den Hartog, 1980) | * | |  |  | | |  |
| *Isarachnanthus* cf*. nocturnus* (Hartog, 1977) | * | |  |  | | |  |
| **Platyhelminthes** |  | |  |  | | |  |
| Turbellaria | 252+/-669 | |  |  | | |  |
| **Nemertea** |  | |  |  | | |  |
| Enopla | * | |  |  | | |  |
| **Sipuncula** |  | |  |  | | |  |
| Sipuncula | 15902+/-22550 | |  |  | | |  |
| **Annelida** |  | |  |  | | |  |
| *Dorvillea moniloceras* (Moore, 1909) | 274+/-863 | |  |  | | |  |
| *Harmothoe* sp*.* | 4806+/-12717 | |  |  | | |  |
| *Hermodice carunculata* (Pallas,1766) | 885+/-1193 | |  |  | | |  |
| *Notopygos caribea* Yáñez-Rivera & Carrera-Parra, 2012 | 5946+/-12026 | |  |  | | |  |
| *Phyllohartmania* sp*.* | * | |  |  | | |  |
| Polychaeta sp. 1 | 8579+/-13990 | |  |  | | |  |
| Polychaeta sp. 2 | 252+/-669 | |  |  | | |  |
| Polychaeta sp. 3 |  | | * |  | | |  |
| *Trypanosyllis* sp. 1 | * | |  |  | | |  |
| *Trypanosyllis* sp. 2 | * | |  |  | | |  |
| *Trypanosyllis* sp. 3 | * | |  |  | | |  |
| *Trypanosyllis* sp. 4 | * | |  |  | | |  |
| *Trypanosyllis* sp. 5 | * | |  |  | | |  |
| *Trypanosyllis* sp. 6 | * | |  |  | | |  |
| **Arthropoda** |  | |  |  | | |  |
| *Bahadzia bozanici* Holsinger, 1992 | B | | 1972+/-16318 |  | | |  |
| *Bahadzia* sp. |  | |  |  | | | * |
| *Barbouria yanezi* Mejía, Zarza & López, 2008 |  | |  | B | | |  |
| *Cirolana adriani* Ortiz & Cházaro, 2015 | B | |  |  | | |  |
| *Cymadusa herrerae* Ortiz & Winfield, 2015 | B | |  |  | | |  |
| *Penaeus* sp*.* | 130+/-246 | |  |  | | |  |
| *Leptochelia rapax* Harger, 1879 | B | |  |  | | |  |
| *Janicea antiguensis* (Chace, 1972) | | | B |  | | |  |
| *Mayaweckelia* sp*.* |  | | 5414+/-2688 | 999+/-31 | | |  |
| *Melita longisetosa* Sheridan, 1980 | B | |  |  | | |  |
| *Melita planaterga* Kunkel, 1910 | B | |  |  | | |  |
| *Metacirolana mayana* (Bowman, 1987) | B | | 4146+/-7022 | 67+/-92 | | |  |
| *Parhippolyte sterreri* (C.W.J. Hart & Manning, 1981) |  | | B |  | | |  |
| *Parhyale hawaiensis* (Dana, 1853) | B | |  |  | | |  |
| *Procaris mexicana* von Sternberg & Schotte, 2004 | * | | 225+/-1123 | 178+/-115 | | |  |
| *Pseudopolycope (Pseudopolycope) helix* Kornicker, Iliffe & Harrison-Nelson, 2007 | B | |  |  | | |  |
| *Stenobermuda* sp*.* | * | |  |  | | |  |
| *Stenopus hispidus* (Olivier, 1811) | * | |  |  | | |  |
| *Tulumella unidens* Bowman & Iliffe, 1988 |  | | B |  | | |  |
| *Xibalbanus* sp*.* | B | |  |  | | |  |
| *Xibalbanus cozumelensis* Olesen, Meland, Glenner, van Hengstum & Iliffe, 2017 |  | | * |  | | |  |
| *Yagerocaris cozumel* Kensley, 1988 | B | | B |  | | |  |
| **Mollusca** |  | |  |  | | |  |
| *Ctenoides scaber* (Bron, 1778) | 295+/-689 | |  |  | | |  |
| *Cyclostrema cancellatum* Marryat, 1819 | | | 1945+/-0 |  | | |  |
| *Fugleria tenera* (C. B. Adams, 1845) | * | |  |  | | |  |
| *Isognomon alatus* (Gmelin, 1791) | * | |  |  | | |  |
| *Luria cinerea* (Gmelin, 1791) | * | |  |  | | |  |
| *Macrocypraea zebra* (Linnaeus, 1758) | 270+/-468 | |  |  | | |  |
| Heterobranchia | 39+/-92 | |  |  | | |  |
| *Volvarina avena* (Kiener, 1834) | 44168+/-32145 | |  |  | | |  |
| **Echinodermata** |  | |  |  | | |  |
| *Amphipholis* cf*. squamata* | * | |  |  | | |  |
| *Asterinides* sp. | 256+/-341 | | B |  | | |  |
| *Astropecten duplicatus* Gray, 1840 | B | |  |  | | |  |
| *Brissopsis* cf*. atlantica* | * | | * |  | | |  |
| *Copidaster cavernicola* Solis-Marin & Laguarda-Figueras, 2010 | 110+/-178 | |  |  | | |  |
| *Diadema antillarum* Philippi, 1845 | B | |  |  | | |  |
| *Euapta lappa* (J. Müller, 1850) | * | |  |  | | |  |
| *Eucidaris tribuloides* (Lamarck, 1816) | 270+/-468 | |  |  | | |  |
| *Holothuria (Semperothuria) surinamensis* Ludwing, 1875 | 123+/-213 | |  |  | | |  |
| *Lytechinus* sp*.* | B | |  |  | | |  |
| *Meoma ventricosa* (Lamarck, 1816) | * | |  |  | | |  |
| *Mithrodia clavigera* (Lamarck, 1816) | 147+/-511 | |  |  | | |  |
| *Ophiactis algicola* H.L. Clark, 1933 | * | |  |  | | |  |
| *Ophiocoma wendtii* Müller & Troschel, 1842 | 36+/-116 | |  |  | | |  |
| *Ophioderma brevispina* (Say, 1825) | * | |  |  | | |  |
| *Ophioderma appressa* (Say, 1825) | * | |  |  | | |  |
| *Ophioderma ensifera* Hendler & Miller, 1984 | B | |  |  | | |  |
| *Ophiolepis* cf*. impressa* | * | |  |  | | |  |
| *Ophiomusa* cf*. testudo* | 13021+/-16478 | |  |  | | |  |
| *Ophionereis* cf*. reticulata* | 268365+/-131735 | |  |  | | |  |
| *Ophiothrix (Ophiothrix) angulata* (Say, 1825) | 905+/-2136 | |  |  | | |  |
| *Ophiothrix brachyactis* H.L. Clark, 1915 | * | |  |  | | |  |
| *Ophiothrix lineata* Lyman, 1860 | * | |  |  | | |  |
| *Ophiothrix (Ophiothrix) oerstedii* Lütken, 1856 | 221+/-744 | |  |  | | |  |
| *Ophiothrix (Acanthophiothrix) suensonii* Lütken, 1856 | 110+/-350 | |  |  | | |  |
| *Ophiura* cf*. ljungmani* | * | |  |  | | |  |
| **Chordata** |  | |  |  | | |  |
| *Typhliasina pearsei* (Hubbs, 1938) | 252+/-669 | | 648+/-391 |  | | |  |
| *Didemnum* sp. |  | | 71150+/-13234 |  | | |  |
| *Pyura* cf*. munita* | 4006+/-5909 | |  |  | | |  |
| *Ascidia* sp. 1 | 12307+/-22865 | |  |  | | |  |
| *Ascidia* sp. 2 | 295+/-722 | |  |  | | |  |
