# Supplementary material for: Ecological patterns in anchialine caves
Source: PLoS One. 2018 Nov 7;13(11):e0202909. doi: 10.1371/journal.pone.0202909 (PMC6221257; doi:10.1371/journal.pone.0202909)
Supplement: S4 Table — (DOCX) [file pone.0202909.s007.docx]

**S4 Table. – *A priori* and *a posteriori* statistics**

Kolmogorov-Smirnov (α=0.05) normality test. Significant differences are shown in red.

| Cave | Site | N | max D | p |
| --- | --- | --- | --- | --- |
| El Aerolito | a | 27 | 0.36791 | p < .01 |
|  | b | 108 | 0.28274 | p < .01 |
|  | c | 90 | 0.36362 | p < .01 |
|  | d | 108 | 0.38856 | p < .01 |
| La Quebrada | a | 12 | 0.24056 | p > .20 |
|  | b | 12 | 0.35763 | p < .10 |
|  | c | 12 | 0.38317 | p < .05 |

*A posteriori* test of multiple comparisons of mean ranks for all groups for Kruskal-Wallis of El Aerolito Cave. Significant differences are shown in red.

|  | a | b | c | d |
| --- | --- | --- | --- | --- |
| a | - | - | - | - |
| b | p > .01 | - | - | - |
| c | p > .01 | p > .01 | - | - |
| d | p > .01 | p < .01 | p < .01 | - |
