# Supplementary material for: Ecological patterns in anchialine caves
Source: PLoS One. 2018 Nov 7;13(11):e0202909. doi: 10.1371/journal.pone.0202909 (PMC6221257; doi:10.1371/journal.pone.0202909)
Supplement: S5 Table — (DOCX) [file pone.0202909.s008.docx]

**S5 Table. - Specific density (org/m^2^) per surveyed cave site (SD: standard deviation).**

|  |  | **Site "a"** | | **Site "b"** | | **Site "c"** | | **Site "d"** | |
| --- | --- | --- | --- | --- | --- | --- | --- | --- | --- |
| **Cave** | **Specie** | Density | SD | Density | SD | Density | SD | Density | SD |
| **El Aerolito** | *Acarnus innominatus* | 0.022 | 0.038 | 0.022 | 0.052 |  |  | 0.122 | 0.243 |
|  | Agelasidae | 0.133 | 0.133 | 0.350 | 0.700 |  |  | 0.106 | 0.104 |
|  | Calcarea sp. 2 | 1.933 | 1.562 | 0.078 | 0.188 | 0.173 | 0.269 | 0.128 | 0.196 |
|  | Calcarea sp. 4 | 0.067 | 0.115 |  |  |  |  |  |  |
|  | Chondrosiida |  |  | 0.017 | 0.033 |  |  |  |  |
|  | *Diplastrella megastellata* |  |  | 0.244 | 0.397 | 0.067 | 0.133 | 0.139 | 0.146 |
|  | *Gastrophanella* sp*.* & *Aciculites higginsii* | 0.022 | 0.038 | 0.047 | 0.071 | 0.015 | 0.044 | 0.047 | 0.055 |
|  | *Geodia neptuni* | 0.711 | 0.668 | 0.339 | 0.253 | 0.140 | 0.173 | 0.039 | 0.053 |
|  | *Geodia* sp*.* 1 |  |  | 0.167 | 0.167 | 0.060 | 0.086 | 0.178 | 0.237 |
|  | *Geodia* sp. 2 | 0.089 | 0.102 | 0.061 | 0.125 |  |  | 0.100 | 0.227 |
|  | Haliclona (Reniera) sp. 1 | 0.044 | 0.038 |  |  |  |  |  |  |
|  | *Placospongia* sp*.* |  |  | 0.160 | 0.153 | 0.156 | 0.167 | 0.010 | 0.025 |
|  | *Plakortis angulospiculatus* | 0.044 | 0.038 |  |  |  |  |  |  |
|  | *Plakortis* sp*.* |  |  | 0.017 | 0.033 |  |  |  |  |
|  | *Stelletta* sp*.* |  |  |  |  |  |  | 0.011 | 0.026 |
|  | *Tethya* sp. 1 & sp. 2 |  |  | 0.029 | 0.052 | 0.267 | 0.400 | 0.010 | 0.025 |
|  | Actiniaria | 0.022 | 0.038 |  |  |  |  |  |  |
|  | *Balanophyllia (Balanophyllia) bayeri* |  |  | 0.150 | 0.300 |  |  |  |  |
|  | Turbellaria |  |  | 0.010 | 0.025 |  |  |  |  |
|  | Sipuncula |  |  | 0.567 | 0.780 | 0.153 | 0.331 |  |  |
|  | *Dorvillea moniloceras* | 0.011 | 0.027 | 0.004 | 0.015 | 0.008 | 0.023 |  |  |
|  | *Harmothoe* sp. |  |  | 0.181 | 0.479 |  |  |  |  |
|  | *Hermodice carunculata* |  |  | 0.033 | 0.045 |  |  |  |  |
|  | *Notopygos caribea* | 0.022 | 0.038 | 0.211 | 0.428 | 0.007 | 0.021 | 0.006 | 0.019 |
|  | *Polychaeta* sp. | 0.067 | 0.067 | 0.289 | 0.488 | 0.013 | 0.028 | 0.006 | 0.019 |
|  | *Polychaeta* sp. 2 |  |  | 0.010 | 0.025 |  |  |  |  |
|  | *Penaeus* sp. |  |  |  |  | 0.020 | 0.032 | 0.006 | 0.019 |
|  | *Ctenoides scaber* |  |  | 0.011 | 0.026 |  |  |  |  |
|  | *Cypraea zebra* | 0.022 | 0.038 |  |  |  |  |  |  |
|  | Heterobranchia |  |  |  |  |  |  | 0.011 | 0.026 |
|  | *Volvarina avena* | 0.333 | 0.240 | 1.317 | 0.927 | 0.927 | 0.828 |  |  |
|  | *Asterinides* sp. |  |  |  |  |  |  | 0.072 | 0.096 |
|  | *Copidaster cavernicola* |  |  |  |  | 0.020 | 0.032 |  |  |
|  | *Eucidaris tribuloides* | 0.022 | 0.038 |  |  |  |  |  |  |
|  | *Holothuria (Semperothuria) surinamensis* |  |  |  |  | 0.022 | 0.038 |  |  |
|  | *Mithrodia clavigera* |  |  | 0.006 | 0.019 |  |  |  |  |
|  | *Ofionereis reticulata* | 6.711 | 2.655 | 4.578 | 2.113 | 11.533 | 7.490 | 0.328 | 0.502 |
|  | *Ophiocoma wendtii* |  |  |  |  | 0.007 | 0.021 |  |  |
|  | *Ophiomusa* cf*. testudo* | 0.022 | 0.038 | 0.480 | 0.603 |  |  |  |  |
|  | *Ophiothrix (Ophiothrix) angulata* |  |  | 0.033 | 0.078 |  |  | 0.006 | 0.019 |
|  | *Ophiothrix (Ophiothrix) oerstedii* |  |  | 0.006 | 0.019 | 0.013 | 0.042 |  |  |
|  | *Ophiothrix (Acanthophiothrix) suensonii* |  |  |  |  | 0.020 | 0.063 |  |  |
|  | *Ascidia* sp. 1 | 0.044 | 0.077 | 0.413 | 0.784 | 0.133 | 0.180 | 0.013 | 0.028 |
|  | *Ascidia* sp. 2 |  |  | 0.011 | 0.027 |  |  |  |  |
|  | *Pyura* cf. *munita* | 0.022 | 0.038 | 0.094 | 0.149 | 0.200 | 0.245 | 0.033 | 0.035 |
|  | *Typhliasina pearsei* |  |  | 0.010 | 0.025 |  |  |  |  |
| **La Quebrada** | *Diplastrella* sp. | 0.200 | 0.067 |  |  | 0.022 | 0.038 |  |  |
|  | *Discodermia adhaerens* | 0.044 | 0.077 |  |  |  |  |  |  |
|  | *Haliclona (Reniera)* sp. 2 | 0.311 | 0.192 |  |  | 0.022 | 0.038 |  |  |
|  | *Bahadzia bozanici* |  |  | 0.556 | 0.559 |  |  |  |  |
|  | *Mayaweckelia* sp. | 0.178 | 0.154 |  |  | 0.022 | 0.038 |  |  |
|  | *Metacirolana mayana* |  |  | 0.089 | 0.102 | 0.111 | 0.139 |  |  |
|  | *Procaris mexicana* |  |  | 0.022 | 0.038 |  |  |  |  |
|  | *Cyclostrema cancellatum* |  |  |  |  | 0.067 | 0.000 |  |  |
|  | *Didemnum* sp. | 1.689 | 0.749 | 0.044 | 0.077 | 0.733 | 0.115 |  |  |
|  | *Typhliasina pearsei* | 0.022 | 0.038 |  |  |  |  |  |  |
| **Tres Potrillos** | *Mayaweckelia* sp. | 0.333 | 0.067 | 0.033 | 0.047 |  |  |  |  |
|  | *Metacirolana mayana* | 0.156 | 0.214 | 0.033 | 0.047 |  |  |  |  |
|  | *Procaris* sp. | 0.422 | 0.269 | 0.033 | 0.047 |  |  |  |  |
